# Advances in Antiviral Drug Development Targeting Enteroviruses: From Viral Proteins to Host Factors

**DOI:** 10.3390/v18040476

**Published:** 2026-04-18

**Authors:** Jiaying Lu, Congyi Li, Wenzhe Cui, Yining Du, Jiayi Geng, Wenyan Zhang

**Affiliations:** Key Laboratory of Organ Regeneration and Transplantation of the Ministry of Education, Institute of Virology and AIDS Research, Center of Infectious Diseases and Pathogen Biology, The First Hospital of Jilin University, Changchun 130021, China; ljy6775@163.com (J.L.); congyi25@mails.jlu.edu.cn (C.L.); cuiwz21@mails.jlu.edu.cn (W.C.); du_yi_ning@163.com (Y.D.); gengjy25@mails.jlu.edu.cn (J.G.)

**Keywords:** enterovirus, hand-foot-mouth disease, antiviral

## Abstract

Enteroviruses represent important human pathogens, posing a substantial disease burden, particularly in children under 5 years of age. Enteroviruses are the primary causative agents of hand-foot-and-mouth disease (HFMD) and are strongly associated with acute flaccid myelitis (AFM), with severe cases potentially resulting in significant neurological complications. Inactivated vaccines against EV-A71 based on the C4 genotype are currently available. However, there are no licensed direct antiviral agents for severe cases. By focusing on viral proteins and host factors, researchers have made great strides in the creation of antiviral medications that target enteroviruses. However, several viral candidates failed to progress in clinical development due to limited efficacy or side effects. This review discusses key findings in enterovirus antiviral research, analyzes the advantages and limitations of each drug target, and highlights knowledge gaps that need to be addressed to advance further development in this field.

## 1. Introduction

Enteroviruses (EVs) belong to the genus Enterovirus within the family Picornaviridae. According to the current classification by the International Committee on Taxonomy of Viruses (ICTV), the genus Enterovirus comprises 15 species, designated Enterovirus A–L and Rhinovirus A–C [1]. Among these, seven species infect humans, including Enterovirus A–D and Rhinovirus A–C [2,3]. Human enteroviruses encompass numerous types such as polioviruses, coxsackieviruses A and B, echoviruses, and several numbered enteroviruses that are classified based on VP1 sequence identity. The classification of human enteroviruses is shown in Table 1.

Enteroviruses are non-enveloped, positive-sense, single-stranded RNA viruses containing approximately 7500 nucleotides. The viral particles consist of an icosahedral capsid with a diameter of about 30 nm [4]. The EV-A71 strain was first isolated in California in 1969 [5]. Since then, outbreaks of hand-foot-and-mouth disease (HFMD) caused by EV-A71 have occurred frequently worldwide, making EV-A71 infection a major public health issue. This infection primarily occurred in Europe and Asia, including Spain [6], Japan, and China [7,8], and resulted in a significant number of deaths. In addition to EV-A71, CV-A6 and CV-A16 are also major sources of HFMD infection [9,10]. Enterovirus infections are typically mild, but children under 5 years of age are particularly susceptible to the most severe neurological diseases associated with EV-A71, including aseptic meningitis, brainstem and/or cerebellar encephalitis, and acute flaccid paralysis (AFP) [11,12]. The pathogenesis of HFMD remains unclear, and it is primarily transmitted through the fecal–oral route, respiratory droplets, and close contact [13]. In addition to the aforementioned viruses, EV-D68 is a special enterovirus that mainly causes severe respiratory disease and neurological disorder, acute flaccid myelitis (AFM) [14]. A recent study showed that the clinical severity of HFMD was positively correlated with EV-A71 viral genome load in throat swabs [15]. It also suggests that developing an antiviral drug to lower viral loads and alleviate clinical symptoms is an effort researchers should make.

**Figure 1:**
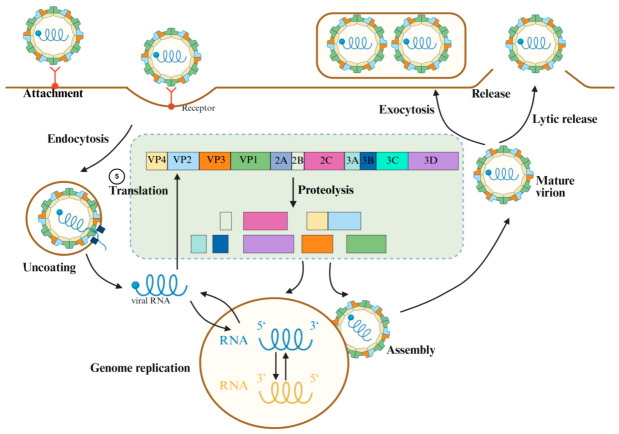
Life cycle of enteroviruses.

For the development of antiviral drugs, understanding the structure and life cycle of viruses and identifying potential drug targets is crucial [16]. The EVs’ genome primarily consists of the following segments, arranged from the 5′ end to the 3′ end: the 5′ untranslated region (5′ UTR), the open reading frame (ORF), the 3′ UTR, and a variable-length polyadenylate tail (poly A) [17]. The ORF encodes a polyprotein that can be cleaved into three functional domains: P1, P2, and P3, each playing an indispensable role in the viral life cycle. The structural protein P1 resides at the N-terminus of the polyprotein, encoding four viral capsid proteins: VP1, VP2, VP3, and VP4. The non-structural proteins P2 and P3 are cleaved into seven distinct proteins—2A, 2B, 2C, 3A, 3B, 3C, and 3D—which collectively form the primary enzymes essential for viral survival [18]. The replication cycle of EVs can be primarily divided into the following steps: attachment, endocytosis, uncoating, genome replication, translation, assembly, mature virion, and release (Figure 1). In response to the aforementioned lifecycle, scientists have made numerous efforts to prevent and control EVs. Currently, China has approved three EV-A71 vaccines for commercial use, and a DNA-based tetravalent vaccine (VP1me) targeting EV-A71, CV-A16, CV-A10, and CV-A6 VP1 proteins [19,20]. However, there remains a lack of specific antiviral drugs for clinical use, with treatment primarily relying on symptomatic and supportive care. Although some existing drugs and investigational new drugs offer therapeutic options from different angles, most have specific indications and limitations. To date, no FDA-approved drug for EVs has been marketed. Therefore, the development of novel drugs remains the top priority in EV treatment.

Antiviral drugs can be categorized into three types depending on their chemical structure: small molecules, peptides, and protein-based therapies. The most common and widely used type of antivirals is small molecules. This study excludes drugs with unclear or conflicting activities and instead looks at small-molecule antiviral candidates that target EVs and have proven mechanisms of action or shown in vivo antiviral effectiveness in animal models. We employ the same nomenclature as the original publications for compounds that are cited in this study.

## 2. Ascertained Protein Targets

### 2.1. Capsid Inhibitors

EV virus particles contain a single-stranded positive-sense RNA genome surrounded by an icosahedral capsid composed of viral capsid proteins VP1, VP2, VP3, and VP4. VP1, VP2, and VP3 form the outer surface of the capsid, whereas VP4 is located internally [21]. A canyon-like depression surrounding the five-fold axis of the capsid, mainly formed by VP1 together with VP2 and VP3, serves as a receptor-binding region. This canyon can interact with multiple cellular receptors, including human scavenger receptor B-like 2 (hSCARB2) [22], human p-selectin glycoprotein ligand 1 (PSGL-1) [23], annexin a 2 (AnxA2) [24], heparan sulfate [25], sialylated glycans [26] and the dendritic cell intercellular adhesion molecule-3 non-integrin (DC-SIGN) [27], thereby facilitating viral attachment and entry. Targeting the capsid proteins, particularly VP1, has become an effective antiviral strategy. Capsid-binding compounds can block receptor attachment, stabilize the viral capsid, prevent uncoating, and inhibit the release of viral RNA.

Among the earliest potential treatments for viral infections were VP1 inhibitors [28]. Pleconaril has been recognized as a chemical that selectively binds to the VP1 capsid protein of enteroviruses, causing a conformational alteration in the VP1 protein. This interferes with the replication process after viral entry into host cells, thereby inhibiting enterovirus infection [29,30]. In addition, drugs such as vapendavir and pocapavir have also been shown to be somewhat effective against enterovirus infections [31]. Nevertheless, not all viral infections can be treated with these drugs. Research on EV-D68 demonstrated that vapendavir suppressed several strains of EV-D68, although only one exhibited single-to sub-micromolar efficacy [32]. Conversely, another study showed it was inactive against all four tested EV-D68 strains. With a low selectivity index, pirodavir showed little action against EV-D68 [33]. This may be due to differences in the VP1 sequence of the capsid inhibitor drug-binding site. The most prevalent strategy for addressing these issues and mitigating drug resistance is the combination of pharmaceuticals with several mechanisms of action. Aleksandr Ianevski et al. tested pleconaril, rupintrivir, and remdesivir in combination on human lung epithelial A549 cells. They found that compared to either monotherapy or dual therapy, combination therapy was substantially more successful against CV-B5 and other viruses. Their study demonstrates how combination drugs can significantly lower the risk of drug-resistant virus strains while also improving treatment efficacy [34]. V-073 is a small-molecule anti-polio inhibitor, while BTA-798 is a pirodavir analog exhibiting significant activity against human rhinoviruses A and B groups and multiple enteroviruses [35]. Additionally, there is a notable synergistic antiviral impact when these two drugs are combined [36].

Tanomastat is a newly identified substance that has notable efficacy against EV types A, B, C, and D in vitro and exhibits dose-dependent inhibitory effects on EV replication. Tanomastat mainly interferes with the initial phases of the replication cycle of EV-A71 [37]. Mechanistically, tanomastat specifically prevents viral capsid dissociation by binding to the VP1 hydrophobic pocket.

Furthermore, certain capsid inhibitor-derived small-molecule drugs have antiviral properties as well. Through its interaction with VP1, the imidazolidinone derivative (PR 66) prevents EV infection. Potent structural analogs of PR66, such as NLD and ALD, have been developed with IC_50_ values of 0.025 and 8.54 nM, respectively [38]. Isoxazole-3-carboxamide (11526092) demonstrated potent in vitro antiviral activity [39]. In a mouse respiratory model infected with EV-D68, this compound exhibited promising antiviral activity against EV-D68. Then, using cryo-electron microscopy (cryo-EM), scientists showed that 11526092 attaches to EV-D68 VP1 from the MO strain in a manner similar to pleconaril, revealing a binding mode distinct from that observed for pleconaril in the Fermon and MO strains. In addition, researchers also found that 11526092 has an effective inhibitory effect on CV-B3 and CV-B5. In a mouse model infected with CV-B5, testing revealed a 3-log decrease in pancreatic TCID_50_. By attaching itself to the hydrophobic pocket of the viral capsid protein VP1, the tetrazole drug R856932 inhibits a number of modern EV-D68 strains with single-digit to sub-micromolar efficacy, blocking viral uncoating and the release of the viral RNA within infected cells [40]. G197 is an active capsid-binding inhibitor against EV-A71. It is a structurally chimeric design, derived from two known capsid inhibitors, namely BPROZ-194 and vapendavir. The authors conducted in vitro antiviral assays on G197, confirming its antiviral efficacy, not only against EV-A71 but also demonstrating significant inhibitory effects against CV-A6 and CV-A16 [41].

Some small-molecule compounds from traditional Chinese medicine also have a good inhibitory effect. Dingran Zhao [42] et al. initially identified magnolol as an active substance against EV-A71 by screening a variety of small-molecule compounds. Magnolol is a bioactive component extracted from the traditional Chinese medicine Magnolia officinalis. By reducing the expression of EV-A71 virus VP1 protein, Magnolol significantly reduces the production of EV-A71 virus particles, thereby inhibiting viral replication. It also showed broad-spectrum antiviral activity against CV-B 3, CV-B 4-5, CV-B 4-7, and ECHO-11. Specifically, magnolol activates nuclear factor erythroid 2-related factor 2 (Nrf2), leading to upregulation of the cystine transporter SLC7A11 and increased glutathione (GSH) synthesis. Elevated GSH levels reduce oxidative stress and reactive oxygen species (ROS) production, thereby inhibiting EV-A71 replication. These findings suggest that magnolol suppresses enterovirus infection by targeting the Nrf2–SLC7A11–GSH signaling pathway.

Except for small-molecule drugs, peptide medications that target enterovirus VP1–VP4—particularly VP1—represent another promising antiviral approach. SP40 stabilizes the viral capsid and prevents the conformational shift required for infection by firmly binding to the GH loop of VP1. Viral attachment to the SCARB2 receptor and the ensuing uncoating processes are hence inhibited [43,44]. Furthermore, studies have shown that the greatest degree of viral suppression is achieved when both SCARB2 and the SP40 peptide are blocked together.

In addition to compounds targeting VP1, compounds targeting other parts of the capsid protein have also been developed in current research. Platelet-derived factor 4 (PF4) has been demonstrated to regulate multiple viral infections, and PF4 is a potent entry inhibitor for EV-A71 and CA-16. It exerts its effects by binding to the VP3 protein of EV-A71 and CV-A16 or by interacting with the receptor SCARB2 [45]. Subsequently, researchers reported that the 15-amino acid C-terminal peptide C15 of PF4, including its mutants C15M and C15A, specifically binds to the VP3 capsid protein of CA-6 and EV-D68, thereby disrupting their attachment to host cell surfaces [46]. Furthermore, the VP3 structure of EVs contains a conserved domain critical for C15 interaction. This domain harbors an aspartic acid residue that confers a net negative charge; replacing this residue with a neutral amino acid reduces VP3’s binding affinity for C15. This finding provides additional corroboration for PF4’s anti-enterovirus activity.

At present, scientists have made a lot of efforts in the development of compounds/drugs targeting capsid proteins. Despite the current challenges of drug resistance, through drug structure optimization and the combination of drugs with other mechanisms of action, drug resistance has become a major concern; they remain a promising weapon in the treatment of life-threatening enteric viral infections, such as neonatal and neurological infections. Future research and development will continue to focus on developing safer and more effective capsid inhibitors and incorporating them into combination therapy regimens. However, because these drugs often need to be used early in the infection to be most effective, early diagnosis is often difficult, and this is one of the difficulties in developing drugs that target VP1. Peptide drugs block viral entry by mimicking receptors or directly binding to the capsid, with a unique mechanism. Despite the challenges in stability and delivery, with the advancement of polypeptide modification technologies (e.g., introduction of D-type amino acids, cyclization, pegylation), more and more studies have focused on the modification of peptides. Such drugs are expected to be effective in the prevention or treatment of severe enteroviral infections, such as neonatal and neurological infections. This is especially the case with topical agents (e.g., nasal sprays, inhalers) or intravenous agents used to treat systemic infections.

**Figure 2:**
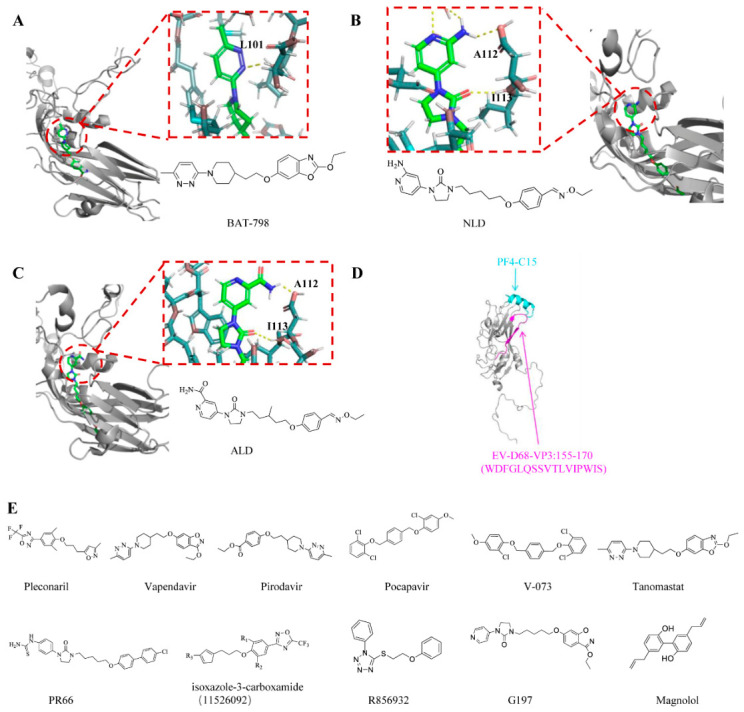
In panels (**A**–**C**), nitrogen and oxygen atoms are shown in blue and red, respectively, and the inhibitor carbon atoms are shown in green. (**A**) Crystal structure of HRV2 VP1 complexed with compound BTA798 (**6**) (PDB:3VDD) and the chemical structure of BTA798. (**B**) Crystal structure of EV-A71 VP1 complexed with inhibitor NLD (**9**) (PDB:4CEY) and the chemical structure of NLD. (**C**) Crystal structure of EV-A71 VP1 complexed with inhibitor ALD (**10**) (PDB:4CEW) and the chemical structure of ALD. (**D**) Protein structure of PF4-C15 (PDB:1WAB). (**E**) Chemical structural formula of the EVs capsid protein inhibitor.

Figure 2 shows the chemical structures of all small-molecule compounds, as well as interaction models between some of these compounds and capsid inhibitors.

### 2.2. 2A Pro Inhibitors

The 2Apro of EVs is a viral cysteine protease that cleaves viral polyproteins between the P1 and P2 segments. In addition, 2Apro can also cleave eIF4G of host cells, inhibiting host cap-dependent mRNA translation [47]. Wang et al. discovered that the His-Asp-Cys catalytic triad and Ser/thr125 are highly conserved in enteroviruses and are crucial locations for the creation of broad-spectrum antiviral medications [48]. However, no licensed drugs with demonstrated efficacy and specificity against EV infections have surfaced because the 2Apro is still a largely unexplored therapeutic target.

Research has found that a hexapeptide (LVLQTM) can effectively inhibit the cleavage activity of the EV-A71 2Apro protein and the replication process of EV-A71 [49]. Researchers infected HeLa cells with EV-A71 and treated them with 200 μM z-LVLQTM-factors at different post-infection time points. They observed that administering the peptide at 2 and 4 h post-infection produced more pronounced inhibitory effects on viral replication. Similar to LVLQTM, extracts from Schizonepeta and Melissa officinalis inhibit CAP-dependent translation initiation by preventing 2Apro-mediated cleavage of eIF4G. They also suppress hnRNP A1 transport and ROS-induced p38 kinase activation, leading to multi-target inhibition of EV-A71 [50]. Speckle-type POZ protein (SPOP) acts as a host E3 ubiquitin ligase that triggers EV-A71-2Apro ubiquitination modification and degradation [51]. SPOP promotes lysosome-dependent degradation of EV-A71-2Apro by inducing K48-linked polyubiquitination of EV-A71-2Apro, ultimately limiting EV-A71 replication.

In a study of small-molecule inhibitors, the investigators identified CW-33 as a weak inhibitor of EV-A71 2Apro [52]. It inhibited the cleavage of IFNAR1 mediated by 2Apro and restores the phosphorylation of Tyk2 and STAT1 induced by type I IFN in EV-A71-infected cells, as well as the upregulation of 2′,5′-OAS. Meanwhile, researchers also found that the anti-EV-A71 activity of CW-33, in combination with IFN-β, showed synergistic efficacy in plaque reduction and virus yield inhibition experiments. This also suggests that CW-33, in combination with low doses of type I IFN, could be used to develop alternative treatments for EV-A71 infection. Furthermore, in studies of other small-molecule inhibitors, (5-oxazolyl) phenyl amine derivatives were found to show strong activity against CV-B 3 and/or CV-B 6 at low concentrations (IC_50_ < 2.0 μM) [53].

Telaprevir is a strong antiviral drug when abiding by an FDA-approved regimen for Hepatitis C Virus (HCV) infected disease. In the context of the old-drug new-use strategy, telaprevir was shown to inhibit EV-D68 2Apro through a near-irreversible biphasic mechanism. Telaprevir was low micromolar to sub-micromolar in cell culture. Treatment with telaprevir has even been demonstrated in animal studies to protect the population of motor neurons and lessen weakness in the limbs of animals other than the injected hindlimb [54,55]. The chemical Jun11762 was created by scientists building on this basis [56]. Through structure-based guided optimization, telaprevir was converted into Jun11762, an improved 2Apro inhibitor. In Jun11762, cyclohexyl (β-branched) was substituted for norvaline, the P1 residue of telaprevir. This alteration improves binding affinity, decreases cytotoxicity, and strengthens hydrophobic interactions. According to MD simulations, Jun11762 has stronger hydrophobic contacts with I128 and creates an extra hydrogen bond between P1 and H18 than telaprevir.

The 2A protein has comparatively fewer mutations than other antiviral medication targets. As a result, in addition to their potential broad-spectrum antiviral effectiveness, inhibitors that target 2A also have a decreased risk of resistance development. Additionally, drugs that target enterovirus 2A protease can stop this process, improving viral clearance and enabling the host immune system to work more efficiently. This is because enterovirus 2A protease cleaves several host factors to avoid antiviral immune responses. Small-molecule inhibitors and well-thought-out non-cleavable peptide analogs offer a clear path and strong basis for future therapeutic development despite the lack of commercially available medications at this time. In order to overcome resistance issues, future research will concentrate on improving chemical activity, selectivity, and membrane permeability while investigating combination therapy (such as 2Apro inhibitor + RdRp inhibitor).

**Figure 3:**
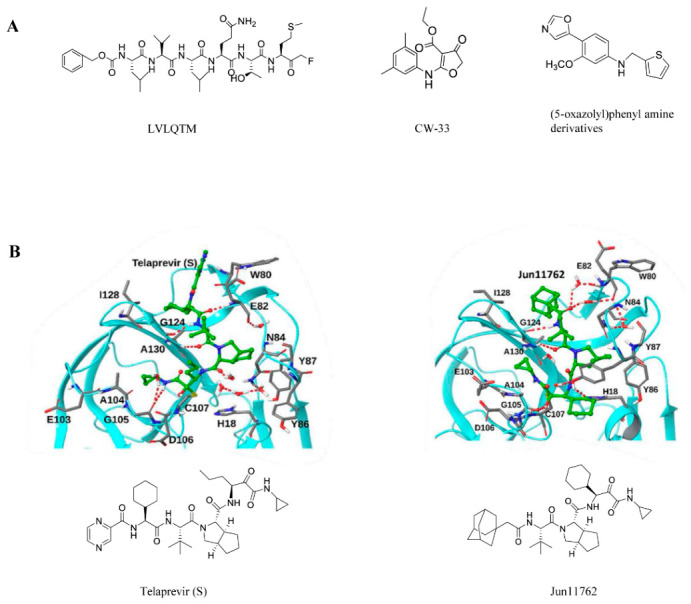
(**A**) Chemical structure of inhibitors targeting 2A protein in EVs. (**B**) Docking model of telaprevir/Jun11762 with EV-D68 2A protease and the chemical structure of telaprevir/Jun11762. (Adapted with permission from ref [56]. Copyright © 2023 American Chemical Society.) In panel (**B**), the ligand is shown in green, with nitrogen and oxygen atoms shown in blue and red, respectively.

Figure 3 shows the structural formulas of all small-molecule compounds that interact with protein 2A, as well as interaction models for some of these compounds and the protein.

### 2.3. 2Bpro Inhibitors

Since the 2B protein is a small transmembrane protein encoded by enteroviruses, primarily functioning within cells and on cell membranes, drug design targeting the 2B protein primarily focuses on inhibiting its pore-forming function or disrupting its interaction with host membranes. EV-A71 2B protein localizes to mitochondria, inducing apoptosis by activating and interacting with the pro-apoptotic protein Bax. In coxsackieviruses, the 2B protein increases ion efflux from the endoplasmic reticulum and Golgi apparatus, which inhibits protein transport through the Golgi apparatus, resulting in inhibition of viral replication. Research indicates that 4,4′-diisothiocyano-2,2′-stilbenedisulfonic acid (DIDS) is a chlorine-dependent current inhibitor that blocks EV-A71 2B activity and leads to inhibition of virus production in RD cells [57]. However, due to DIDS’ structural similarity to many other capsid protein inhibitors, its cellular antiviral activity may extend beyond inhibiting chloride conduction through the 2B ion channel. In addition, Zichun Xiang et al. also found that CD74 can inhibit EV-D68 replication by interacting with the second hydrophobic region of the 2B protein [58].

Therapeutics that target the 2B protein may also become resistant to them as a result of viral alterations, just like other antiviral drugs. Maintaining broad-spectrum activity against several enteroviruses while reducing disruption to regular host cell function is a significant challenge. Furthermore, because the 2B protein primarily operates intracellularly and on the cell membrane, medications must effectively enter cells in order to be effective. Traditional drugs have a hard time reaching these areas; thus, not much research has been conducted in this area.

**Figure 4:**
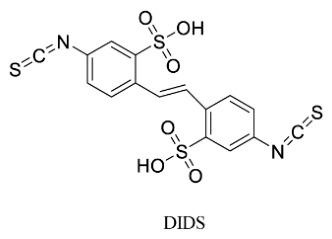
Chemical structure of inhibitors targeting 2B protein in EVs.

Figure 4 shows the chemical structure of DIDS, a small-molecule compound that acts on the 2B protein.

### 2.4. 2Cpro Inhibitors

The enterovirus 2C protein is approximately 329 amino acids in length and contains an N-terminal amphipathic membrane-binding helix, a cysteine-rich zinc-finger motif, an ATPase domain belonging to the AAA+ superfamily, and a C-terminal helical domain [59]. Research shows that 2C ATPase activity and EV viral replication depend on oligomerization [60]. A highly conserved protein that is vital to the enterovirus life cycle is the 2C protein found in EVs. EV 2C has been associated with morphogenesis, RNA replication, host cell membrane rearrangement, ATPase, helicase, chaperone, and virus shelling [61]. The 2C protein also plays a role in controlling the host’s innate immune response after a viral infection. Several small-molecule compounds with a variety of structural variations have been found to be 2C-targeting inhibitors thus far.

In studies of the enterovirus 2C protein, guanidine hydrochloride was the first compound reported to inhibit viral replication through targeting this protein. Later studies identified fluoxetine as another inhibitor of 2C. Both fluoxetine and its metabolite, norfluoxetine, suppress viral replication by preventing the accumulation of viral proteins and RNA. When untreated infected cells and those treated with these chemicals were compared using qPCR analysis, the treated cells’ CV-B3 RNA levels (at the same time point) were significantly lower, by more than 1000 times [62]. Meanwhile, Zuo et al. reported the inhibitory activity of fluoxetine on RNA replication of CV-B1, CV-B2, and CV-B3 (all HEV-B serotypes) [62]. Due to its antidepressant nature, fluoxetine is well-suited to treat AFM induced by EV-D68 and can inhibit multiple EV-D68 strains with micromolar potency in cell culture. Unfortunately, despite its good tolerability and advancement into clinical trials for treating EV-D68 infection, fluoxetine proved ineffective for EV-D68-associated AFM patients [63,64]. Furthermore, fluoxetine exhibited no inhibitory effect against EV-A71 [65]. This also suggests that not all 2C inhibitors have broad-spectrum antiviral activity against enteric viruses. Next, the researchers synthesized several substituted amide compounds and tested them against EVs. The most effective compound, 2C-12b (as indicated in the original document as 12b), exhibited EC_50_ values ranging from 0.0029 to 1.39 μM against multiple enteroviruses, including EV-A71, EV-D68, CV-B3, PV-1, and CV-A24 [66]. Compound 2C-12b is structurally similar to fluoxetine, both containing three aromatic substituents that are linked to the linker. However, unlike fluoxetine, compound 12C-2b has no neuroactivity and does not inhibit the 5-hydroxytryptamine transporter (SERT), dopamine transporter (DAT), or norepinephrine transporter (NET). The mechanism of action was elucidated through resistance screening against CVB3, EV-A71, and EV-D68 viruses. Guanidine inhibits multiple EV-D68 strains in cell cultures with a potency close to 100 μM [67]. Despite its relatively weak in vitro antiviral potency, this compound demonstrated in vivo antiviral efficacy in an EV-D68 infection model. The antiviral mechanism of guanidine was investigated using EV-A71 virus, and it was found that guanidine also had a strong replication-inhibitory effect on EV-A71 [68]. Guanidine comes in a variety of forms, some of which are in the clinical phase and some of which have been approved by the FDA, suggesting that guanidine has some potential for therapeutic development.

Phenylglycine compounds represented by HBB [2-(alpha-hydroxybenzyl)-benzimidazole] are a class of broad-spectrum small-molecule inhibitors against enteroviruses that act directly on the virus itself [69]. These chemicals attach to the GTP/ATP-binding pocket of the 2C protein, competitively inhibiting its ATPase function and obstructing viral RNA replication. Another phenylglycine compound, TBZE-029, is a novel, specific inhibitor against the replication of multiple enteroviruses [70]. The authors’ genotyping of resistant clones revealed three amino acid changes in non-structural protein 2C, specifically at positions 224, 227, and 229, indicating that the compound’s target was located inside the 2C protein.

Dibucaine is an amide-type local anesthetic whose active ingredient is FDA-approved for use in related anesthetic and analgesic products. Rami Musharrafieh and colleagues identified dibucaine as an EV-D68 inhibitor during a drug repurposing screen [67,71]. By blocking sodium channels, dibucaine is utilized as a local anesthetic in clinical settings. However, its clinical use as an antiviral medication is hampered by elements like a low selectivity index and modest antiviral efficacy. As a result, researchers have tried many different approaches to this problem. Concerns over possible adverse effects are allayed by the optimized lead chemical 2C-12A’s lack of sodium channel blockage [67]. Later research showed that 2C-6AW (referred to as 6AW in the original document) had superior in vitro PK characteristics and more extensive antiviral activity, particularly against EV-A71 [72]. In the meantime, another study used structure–activity relationship (SAR) research to establish that dibucaine analogs have a superior cell selectivity index and antiviral activity. The most effective chemical, 2C-6I (formerly listed as 6I), showed in vivo antiviral efficacy and synergistic effects with emetine in a mouse model infected with EV-A71 [73]. Nevertheless, these drugs’ in vitro and in vivo PK characteristics have not yet been documented. Therefore, scientists created three lead compounds—10a, 12a, and 12c—based on structure–activity relationships [67]. All three exhibited significantly improved antiviral efficacy and selectivity indices (EC_50_ < 1 μM, SI > 180). Furthermore, the mechanisms of action for these three compounds were confirmed to be consistent with the 2C protein inhibition mechanism.

Through phenotypic screening, a series of pyrazolopyridine derivatives was identified as antiviral agents against CV-B3. These compounds were subsequently found to exhibit broad-spectrum antiviral activity against several enteroviruses, including poliovirus, coxsackievirus, echovirus, and enterovirus A71 (EV-A71). Mechanistic studies revealed that their antiviral activity is mediated through inhibition of the viral 2C protein. Further structure–activity relationship (SAR) studies led to the development of more potent and selective inhibitors, such as JX040, which inhibits EV-A71 with an EC_50_ value of 0.5 μM [74]. The pharmacokinetic features of this class of drugs, both in vitro and in vivo, require investigation. Jun571 is a pyrazolopyridine-type small molecule identified through high-throughput screening and medicinal chemistry optimization, exhibiting broad-spectrum antiviral activity against enteroviruses. Among these, Jun571 stands out as a lead compound with significant antiviral potency. Subsequently, researchers optimized the structure of Jun571 to obtain Jun6504 [75]. Jun6504 shares the same core skeleton as Jun571, but replaces Jun571’s tertiary dimethylamine side chain with an azetidine-based secondary amine. This modification enhances metabolic stability, alters hydrogen bonding patterns, and significantly improves in vivo pharmacokinetics and therapeutic efficacy.

Other reported 2C inhibitors include MRL-1237, pirlindole, zuclopenthixol, metrifudil, and N6-benzyladenosine [76]. All these compounds were identified from drug repurposing screens, and all have resistance mutations mapping to the viral 2C protein. Although mutations in the viral 2C protein led to resistance to metrifudil and N6-benzyladenosine, no direct binding assays were conducted.

In addition, the development of peptide-based drugs continues to advance. Yuan Fang et al. designed and synthesized the peptide 2CL, targeting the non-structural protein 2C encoded by EVs [77]. This peptide disrupts 2C oligomerization to inhibit its helicase function. It effectively disrupted EV-A71 2C protein oligomerization and suppressed the RNA helicase activity of 2C proteins encoded by EV-A71 and CV-A16. Further studies revealed that 2CL also effectively suppressed EV-A71 replication in vivo, significantly improving the survival rates in mice challenged with EV-A71 and alleviating clinical symptoms in infected mice. Additionally, 2CL demonstrated effective antiviral activity against CV-B3 and ECHO-11, exhibiting broad-spectrum efficacy.

Enterovirus 2C protein is still a highly desired target for antiviral drugs despite our current poor understanding of its mechanisms in viral replication or the host innate immune system. Nevertheless, 2C protein’s intrinsic instability makes it challenging to express itself consistently, which impedes both the development and refinement of inhibitors as well as comprehensive research into its function. Furthermore, the development of structure-based therapy is hampered by the lack of structural evidence on 2C-RNA interactions and the requirement for direct experimental validation of 2C’s function in the replication complex. However, structurally optimized phenethylguanidine compounds and the repurposing of dibucaine are viable strategies. Despite the high specificity of peptide drugs, delivery technology limits their use, necessitating further study. The precise three-dimensional structure of the 2C protein and its several processes must be thoroughly clarified in order to guide sensible medication creation in the future.

**Figure 5:**
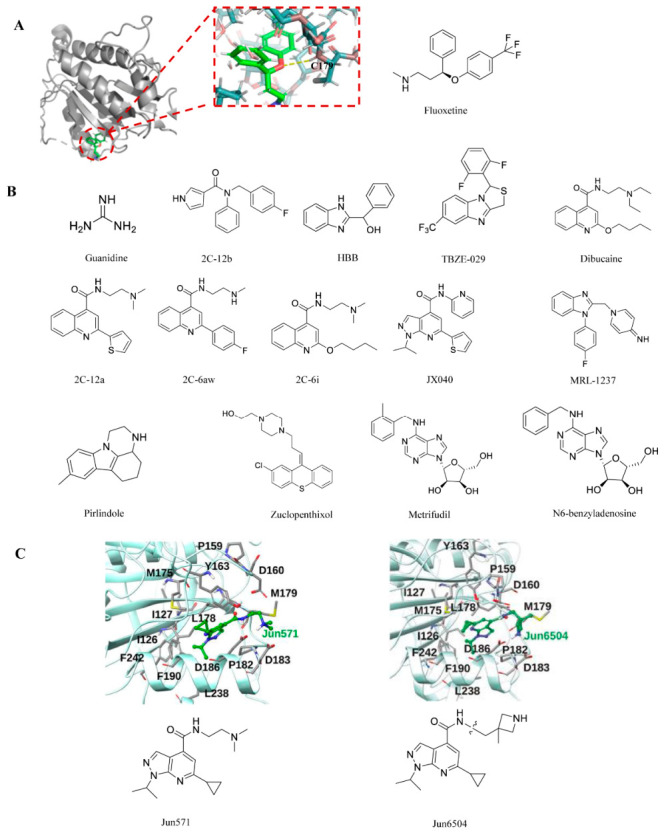
(**A**) Crystal structure of CV-B3 with fluoxetine (PDB:6S3A) and the chemical structure of fluoxetine. (**B**) The chemical structure formula of the inhibitor targeting the 2C protein in EVs. In panel (**B**), the ligand is shown in green, with nitrogen and oxygen atoms shown in blue and red, respectively. (**C**) Structural interactions between EV-D68 2C protein and Jun571/Jun6504, along with chemical structural formulas of compounds. ([75], licensed under CC BY 4.0.).

Figure 5 shows the chemical structures of all small-molecule compounds that interact with the 2C protein, as well as interaction models for some of these compounds with the protein.

### 2.5. 3Apro Inhibitors

The formation of enterovirus replication organelles depends on the viral protein 3A and several host factors, including phosphatidylinositol 4-kinase IIIβ (PI4KB), acyl-CoA-binding domain-containing protein 3 (ACBD3), and oxysterol-binding protein (OSBP). The viral 3A protein recruits ACBD3, which, in turn, mediates the recruitment of PI4KB to viral replication sites. PI4KB generates phosphatidylinositol-4-phosphate (PI4P) lipids that facilitate the formation of replication organelles and viral RNA replication [78,79]. PI4KB, ACBD3, and 3A have been shown to colocalize at viral RNA replication sites. Mutations in 3A, such as I44A or H54Y, disrupt the interaction with ACBD3 and prevent PI4KB recruitment. Further studies by Xiao et al. [80] demonstrated that the ACBD3–PI4KB interaction is essential for the replication of EV-D68.

Enviroxime is one of the earliest 3A protein inhibitors; it achieves this by inhibiting the host PI4KB and disrupting the 3A-mediated recruitment of this kinase to the viral replication membrane [81]. Although early clinical trials have been terminated due to poor oral bioavailability and gastrointestinal side effects, it serves as the precursor compound that provides an important structural and mechanistic basis for the design of subsequent better drugs. As of now, a variety of anti-enterovirus compounds, namely GW5074, AN-12-H5, and flt3 Inhibitor II, are known to harbor similar mutations in the region encoding the 3A protein, resulting in resistance to enviroxime, which is responsible for resistance to enteroviruses, and is regarded as an enviroxime-like compound [82,83]. Novel enviroxime-like compounds, AN-12-H5 and AN-23-F6, can inhibit the early EV-A71 infection after the virus binds to the cells. During the early stage of EV-A71 infection, mutations in the phage proteins were identified as resistance mutations to AN-12-H5 and AN-23-F6. Minetaro Arita et al. analyzed the inhibition of phosphatidylinositol (PI) kinases by previously identified compounds similar to GW5074 and AN-12-H5, along with a newly discovered anti-enterovirus compound, T-00127-HEV1. The investigators found that T-00127-HEV1 inhibited PI4KB activity more specifically than other PI kinases, whereas GW5074 inhibited PI kinases with broad specificity [84]. In contrast, AN-12-H5 had no inhibitory effect on the activity of PI4KB and only a moderate inhibitory effect on the activity of PI3-kinase.

By characterizing the antiviral activity of MDL-860, researchers identified PI4KB as its target. It proved to be a covalent inhibitor by irreversibly modifying C646, located at the bottom of a surface pocket away from the active site. The C646S mutant did not affect the enzymatic activity of PI4KB, suggesting that targeting the allosteric site of C646 localization may not lead to undesired side effects associated with inhibition of the enzymatic activity of PI4KB [85]. This study reveals a novel drug target that can be further explored to develop more effective and specific host-targeting antiviral drugs. Including MDL-860, Adelina Stoyanova et al. proposed a novel antiviral treatment strategy known as consecutive alternating administration (CAA). This method is characterized by the sequential, alternating use of three antiviral drugs—P (pleconaril), M (MDL-860), and O (oxoglaucine)—rather than simultaneous administration. Only one drug is administered per day, with a 3-day cycle repeated for a total of 12 days. CAA therapy significantly improves protection rates and markedly prolongs survival time. Furthermore, CAA not only prevents drug resistance but also synergistically enhances drug efficacy. Compared to the drawbacks of monotherapy—such as the development of drug resistance and a gradual increase in IC_50_—CAA therapy increases viral sensitivity to the drugs [86]. Vemurafenib is an FDA-approved RAF kinase inhibitor for the treatment of BRAFV600 mutation-associated melanoma. In the EV study, vemurafenib was found to work through PI4KB. Concurrently, investigators found that vemurafenib was effective in preventing infection in an acute cell model, eradicating infection in a chronic cell model, and reducing the amount of virus in the pancreas and heart in an acute mouse model [87,88]. Furthermore, CUR-N373 is also a PI4KB inhibitor that suppresses PI4KB activity in vitro and inhibits CV-B4 replication in macrophage cultures [89]. When combined with G197 (a capsid inhibitor), it significantly improves survival rates and reduces pathology in infected mice [41]. Preclinical studies demonstrated that CUR-N373 is effective in both in vitro and in vivo models of EV-A71 infection. Administration of CUR-N373 at a dose of 1 mg/kg to mice improved survival rates in EV-A71-infected mice and significantly reduced the severity of infection, as evidenced by muscle tissue pathology. Since CUR-N373 was observed to have better antiviral properties, the researchers modified it to produce CUR-N399. CUR-N399 is a novel drug developed by Curovir AB that has been evaluated in Phase I clinical trials. It demonstrates improved avoidance of potential drug interactions with concomitant medications and will continue to advance through clinical development [90].

In addition to compounds targeting the host PI4KB protein, numerous compounds targeting the host oxysterol-binding protein (OSBP) have also been developed. Oxysterol-binding protein (OSBP) is a host lipid-transfer protein that mediates the exchange of cholesterol and phosphatidylinositol-4-phosphate (PI4P) between cellular membranes and is essential for enterovirus genome replication. Several small-molecule compounds, such as OSW-1, itraconazole (ITZ), and TTP-8307, inhibit viral replication by targeting OSBP. Among them, OSW-1 is a potent OSBP/ORP4 antagonist that effectively inhibits the replication of enteroviruses [91,92]. TTP-8307 inhibits CV-B3 replication by blocking OSBP-mediated PI4P/cholesterol shuttling and shows cross-resistance with PIK93, a PI4KB inhibitor targeting the host PI4KB-dependent replication pathway [83]. Although resistance mutations have been mapped to the viral 3A protein, no direct binding between TTP-8307 and the 3A protein has yet been demonstrated. Itraconazole (ITZ), a well-known antifungal agent with additional anticancer activity, has also been identified as an EV-A71 inhibitor with an EC_50_ value of approximately 1.15 μM [93,94]. Moreover, ITZ exhibits antiviral activity against several enteroviruses, including CV-A16, CV-B3, and EV-D68, suggesting its potential as a broad-spectrum antiviral agent against enteroviruses.

The enterovirus non-structural protein 3A is a small membrane-associated protein that plays a critical role in viral RNA replication by remodeling intracellular membranes and redirecting host lipid-trafficking pathways. One important host factor recruited during this process is phosphatidylinositol 4-kinase IIIβ (PI4KB). Recruitment of PI4KB promotes the local accumulation of phosphatidylinositol-4-phosphate (PI4P) at viral replication organelles, which is required for the assembly and activity of the viral replication complex. Interfering with this process has therefore attracted considerable interest as a potential antiviral strategy. Several small-molecule compounds, including enviroxime and its derivatives, have been reported to suppress enterovirus replication by targeting the PI4KB pathway and reducing PI4P production at replication membranes. Consistent with this mechanism, resistance mutations to these inhibitors are frequently located in the viral 3A protein. Despite these advances, the sequence variability of 3A among different enterovirus species and the possibility of rapid resistance development continue to present challenges for therapeutic development. Further studies aimed at elucidating the structural basis of 3A–host protein interactions and identifying inhibitors with broader antiviral activity and higher barriers to resistance may facilitate the development of next-generation antivirals targeting this pathway.

**Figure 6:**
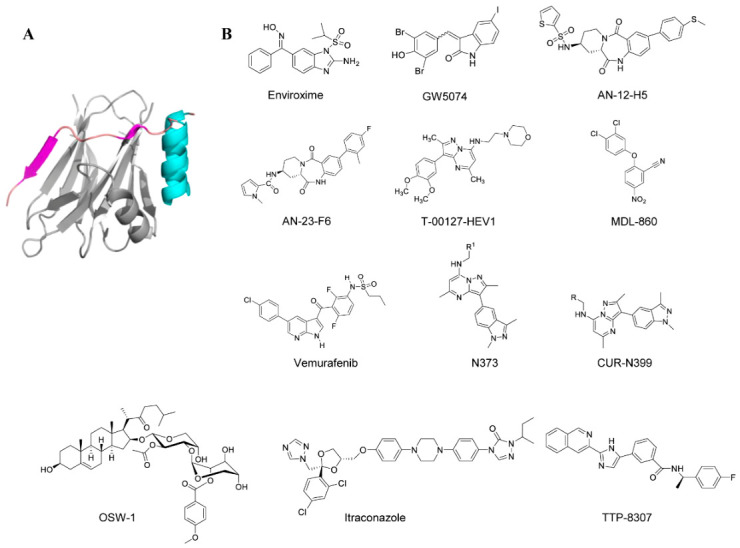
(**A**) X-ray crystal structure of the C-terminal Golgi-dynamics domain (GOLD) of ACBD3 in complex with the cytoplasmic domain of the EV-A71 3A protein (PDB:6HLW). ACBD3 is shown in gray, and the EV-A71 3A protein is shown in a blue-to-purple color scheme. (**B**) Chemical structure of the inhibitor targeting the 3A protein in EVs.

Figure 6 shows the chemical structures of all small-molecule compounds that interact with the 3A protein.

### 2.6. 3Cpro Inhibitors

The 3C (3Cpro) or 3C-like protease (3CLpro) has been extensively studied as an antiviral drug target for foot-and-mouth disease virus [95], norovirus [96], and coronavirus [97]. They are structurally similar to chymotrypsin, though the former is a monomer while the latter is a dimer. In addition to processing several protein precursors, 3Cpro influences toll-like receptors (TLRs), RIG-I-like receptors, Nod-like receptor family PYRIN domain-containing 3 (NLRP3), IFN, and other related signaling pathways by hydrolyzing host proteins. It is essential for host innate immunity and protection against protein expression. Similar to 2Apro, 3Cpro also plays an important role in suppressing host-dependent translation by cleaving eukaryotic initiation factor 4A (eIF 4A) and eukaryotic initiation factor 5B (eIF 5 B). The 3Cpro or 3CLpro exhibits high substrate preference for glutamine at the P1 position, leading most 3C protease inhibitors to be designed with a pyrrolidone moiety at the P1 position as a substrate analog. 3C inhibitors are dipeptides, tripeptides, or tetrapeptides conjugated to reactive warheads. Common reactive warheads include aldehydes, ketoamides, and a, b-unsaturated esters. Aldehyde warheads can also be converted to Bisulfite and cyanohydrin prodrugs [98].

Among drugs targeting EVs 3Cpro, Rupintrivir (AG 7088) is a representative peptide-based compound initially developed as an antiviral agent against human rhinovirus 3C protease. Its antiviral activity has been validated at the molecular, cellular, and individual levels [99]. Nonetheless, the limited oral bioavailability of rupintrivir in naturally infected patients, coupled with apprehensions about administration methods (such as nasal spray) and practical difficulties in real-world infection scenarios, resulted in the drug exhibiting minimal or inadequate clinical advantages in mitigating disease severity or expediting recovery, ultimately prompting its withdrawal from clinical trials. Due to the significant sequence similarity among the 3Cpro or 3CLpro-like proteases of enteroviruses, noroviruses, and coronaviruses, rupintrivir has demonstrated broad-spectrum antiviral efficacy against EV-A71, CV-A16, EV-D68, and noroviruses. Trials against EV-D68 confirmed its potent activity, exhibiting nanomolar potency in cytopathic effect assays [32,100]. Additionally, rupintrivir effectively protected neonatal mice from limb paralysis induced by EV-A71 infection. In fluorescence resonance energy transfer (FRET) assays, its EC_50_ values against EV-A71 and CV-A16 were 0.781 μM and 0.331 μM, respectively [101].

Another analog entering human clinical trials is AG 7404, which has improved oral bioavailability compared with rupintrivir [102]. Eric Rhoden et al. validated that AG 7404 is active against multiple poliovirus strains, with EC_50_ values ranging from 0.080 to 0.674 μM. Moreover, AG 7404 exhibited complete activity against all V-073-resistant variants with EC_50_ values ranging from 0.218 to 0.819 μM, while against V-073-susceptible parent strains, EC_50_ values ranged from 0.202 to 0.407 μM [103]. In vitro combination drug experiments demonstrated synergistic effects between AG 7404 and either V-073 or BTA-798, further substantiating the efficacy of AG 7404. In a cocktail drug research approach, a synergistic combination of pleconaril, AG 7404, and mindeudesivir was discovered that is orally administered and safe for humans, effectively inhibiting the replication of enteroviruses in human cell and organoid cultures [104]. Importantly, this cocktail drug does not alter glucose and insulin levels in pancreatic β-cell cultures and maintains the contraction rhythm of infected cardiac organ tissues (enteroviruses can cause diseases such as myocarditis and type 1 diabetes). These findings highlight a promising cocktail drug for further preclinical studies and clinical trials targeting multiple enterovirus-mediated diseases.

The development of peptidomimetic compounds has remained a key focus for researchers, leading to the discovery of numerous potential inhibitors. Yangyang Zhai et al. designed and synthesized a series of mimetic peptide aldehydes and evaluated their in vitro activity against 3Cpro and EV-A71. Through the study of structure and interrelationships, it was found that Aldehyde 5x exhibited the strongest inhibitory effect on EV-A71 3Cpro (IC_50_ = 0.10 ± 0.02 μM) [105]. NK-1.8 k exhibits the most remarkable antiviral activity (EC_50_ ≈ 0.108 μM) [106], which can inhibit the proliferation of different EV-A71 strains and one strain of EV-D68, and its 50% effective concentration is 90 nM. Low cytotoxicity (50% cytotoxic concentration, >200 μM) indicated a high selective index of over 2000. The 3Cpro inhibitor SG 85 also exhibits excellent antiviral effects (EC_50_ = 180 nM), effectively inhibiting the in vitro replication of 21 EV-A71 virus strains [107,108]. Even the shorter SG75 demonstrated significant anti-rhinovirus activity (EC_50_ = 2–5 μM). An increasing number of compounds have emerged, and their favorable pharmacokinetic properties compared to the parent compound rupintrivir strongly support their development as broad-spectrum antiviral agents.

Another peptidomimetic compound targeting EV-A71 3Cpro is Cyanohydrin (R) -1 [109]. However, due to its cyanohydrin headgroup, cyanohydrin releases cyanide during hydrolysis, leading to stability and potential toxicity issues during drug administration. To improve the reactive warhead, researchers conducted a series of studies on (R)-1. During its modification, they discovered that 4-iminooxazolidin-2-one serves as the bioelectronic isomer of the cyano-alcohol moiety and functions as a doubly activated Michael acceptor. Based on this, scientists designed inhibitors 4e and 4g of 4-iminooxazolidin-2-one. These two compounds inhibited EV-A71 with EC_50_ values of 0.21 and 0.10 μM, respectively, and 4e and 4g significantly enhanced the stability of the drug in human plasma [110]. In addition, the partial structure of 4-iminooxazolidin-2-one with propyl and isopropyl substitutions, namely FOPMC and FIOMC, was also reported. These two compounds can effectively inhibit multiple strains of enteroviruses in various cell lines, and show very little cytotoxicity [111]. In conclusion, 4-iminooxazolidin-2-one partially circumvents the drawbacks of cyanohydrins and is a nonclassical bioisostere replacing cyanohydrin warheads for a wide range of cysteine proteases.

Non-peptide compounds have also contributed to antiviral efficacy, with DC 07090 being a prominent example identified by the researchers through virtual screening. The results showed that it had good inhibitory activity against EV-A71 3C pro with an IC_50_ value of 21.72 ± 0.95 μM and no obvious toxicity (CC_50_ > 200 μM) [112]. It is reported that GC 373, GC 375, and GC 376 can inhibit the replication of EV-A71, with IC_50_ values of 11.1, 15.2, and 10.3 μM, respectively [113]. These three compounds exhibited high inhibitory effects against most tested viruses, with IC_50_ in the high nanomolar or low micromolar range in enzyme-based and/or cell-based assays, and demonstrated high therapeutic indices. A macrocyclic EV-A71 3C protease inhibitor (compound **4**) is designed as an inhibitor of the 3C protease of EV-A71. Its EC_50_ value is 4.5 μM [114].

From compounds screened from natural products, Chenguangyao et al. found that quercetin can inhibit EV-A71-induced cytopathic effects, reduce EV-A71 progeny virus yield, and prevent EV71-induced apoptosis, while exhibiting low toxicity [115]. Further experiments showed that quercetin effectively inhibited the activity of EV-A71 protease 3Cpro, blocking viral replication, without affecting the activity of protease 2Apro or RNA polymerase 3Dpol. Molecular modeling of the 3Cpro-quercetin complex revealed that quercetin is predicted to insert into the substrate-binding pocket of EV-A71 3Cpro, thereby blocking substrate recognition and inhibiting EV-A71 3Cpro activity.

Numerous drug development methods entail the initial identification of a drug candidate, followed by the validation of its mechanism. Recognizing the significance of 3C as a crucial protein in extracellular vesicles, researchers initially designated 3C as a therapeutic target and subsequently developed peptides informed by cleavage sites using functional screening methodologies [116]. Four peptides were developed using the amino acid sequences at extremely efficient cleavage sites for 3C within viral polyproteins. Among the candidates, vp23 had the most powerful action and displayed the highest selectivity index (SI > 185).

Overall, the inhibitors targeting the 3C protein can fundamentally impede viral replication and exhibit broad-spectrum, effective antiviral activity. However, since the in vivo antiviral efficacy of 3Cpro inhibitors in animal models of EV-A71 infection has not been demonstrated, the potential toxic side effects have not been considered. Additionally, a concern for covalent 3Cpro inhibitors is the possibility of off-target consequences [117]. This indicates that 3C-targeting inhibitors require further investigation through multiple follow-up studies by researchers.

**Figure 7:**
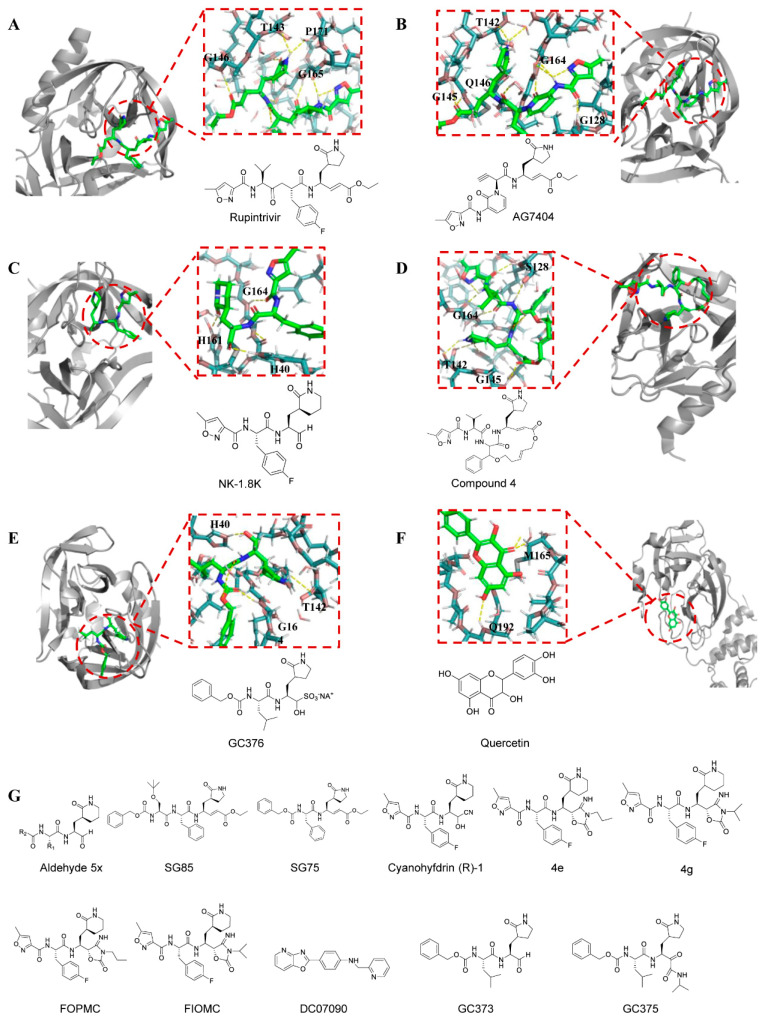
**In panels (**A**–**F**), nitrogen and oxygen atoms are shown in blue and red, respectively, and the inhibitor carbon atoms are shown in green.** (**A**) Crystal structure of the EV-A71 3C protease–rupintrivir complex (PDB:5HXF) and chemical structure of rupintrivir. (**B**) Crystal structures of EV-D68 3C protease in complex with AG7404 (PDB:8W3M) and chemical structure of AG7404. (**C**) Crystal structures of EV-A71 3C protease in complex with NK-1.8k (PDB:5GSO) and chemical structure of NK-1.8k. (**D**) Crystal structure of EV-A71-3C protease with a novel macrocyclic compound (PDB:6LKA) and the chemical structure of compound **4**. (**E**) Crystal structures of EV-D68 3C protease in complex with GC376 (PDB:8W3T) and chemical structure of GC376. (**F**) Crystal structures of EV-D68 3C protease in complex with quercetin (PDB:8GQT) and chemical structure of quercetin. (**G**) Chemical structure of the 3C protein inhibitor for EVs.

Figure 7 primarily shows the molecular formulas of small-molecule compounds that act on the 3C protein, as well as interaction models between some of these compounds and the 3C protein.

### 2.7. 3Dpro Inhibitors

The synthesis of viral RNA is mediated by the RNA-dependent RNA polymerase referred to as the 3D Pol of enteroviruses. At present, the inhibitors targeting 3D Pol are predominantly nucleosides or nucleotide analogs. Three nucleoside compounds—gemcitabine, LY2334737, and sofosbuvir—that demonstrate notable antiviral activity against EV-A71 were found through an analysis of the FDA’s drug library. By targeting the early phases of viral RNA and protein synthesis in EV-A71, gemcitabine considerably lowers infectious EV-A71 titers by 2.5 log PFU/mL. When paired with interferon-β, the inhibition of EV-A71 replication is even more effective [63]. LY2334737 and sofosbuvir also protect mice against the deadly EV-A71 challenge by possibly lowering viral titers, mortality, and virus-induced pathology in mouse limb muscle tissue.

2′-Deoxy-2′-b-fluoro-4′-azidocytidine (FNC) has been demonstrated as a potent inhibitor of HIV. It has completed Phase II clinical trials for treating HIV infection and global Phase III clinical trials for combating COVID-19, and is now commercially available. Additionally, FNC also inhibits replication of a variety of EVs, including EV-A71, CV-A6, CV-A16, EV-D68, and CV-B3 [118]. In vitro, 3D pol activity and isothermal titration calorimetry (ITC) experiments confirmed that FNC inhibited positive- and negative-strand RNA synthesis, mainly by targeting and competitive inhibition of the activity of 3D pol in EV disease. In studies using newborn mouse models infected with EV-A71 and CV-A16, mice treated with 1 mg/kg FNC every two days were protected from virus-induced mortality and exhibited reduced viral loads in various tissues.

Remdesivir (GS-5734) is a novel phosphoramidate adenosine analog prodrug exhibiting potent antiviral activity against multiple RNA virus families. It is also an FDA-approved antiviral drug for SARS-CoV-2 [119,120]. Experiments by Wei Ye et al. showed that remdesivir inhibited EV-A71 viral RNA (vRNA) and complementary RNA (cRNA) synthesis, indicating that the triphosphate (TP) form of remdesivir suppresses EV-A71 replication [121]. Favipiravir (T-705) is a broad-spectrum antiviral drug approved in Japan for the treatment of influenza virus infection [122]. Meanwhile, experiments confirmed its inhibition of EV-A71 replication in cell cultures by targeting viral 3Dpol [123]. A combination of remdesivir and favipiravir showed no antagonistic effects. MRS 7704 also inhibits EV-A71 with an EC_50_ value of 3–4 µM [63]. However, its mechanism of action remains unclear. Similarly to GS-5734, NITD 008 was originally developed as an anti-flavivirus agent. NITD 008 is a viral inhibitor that suppresses viral RNA synthesis, exhibiting inhibitory activity against multiple EV-A71 virus strains in various cell lines. In RD cells, its EC_50_ value is 0.67 μM [124]. When administered at 5 mg/kg in an EV-A71 mouse model, the compound reduced viral load in various organs, linked clinical symptoms, and avoided death from infection [124].

In addition to nucleotide analogs, there are many non-nucleotide analogs that have the same function. DTrip-22 is one of these non-nucleoside inhibitors. During viral infection, this compound inhibits the accumulation levels of both positive-strand and negative-strand viral RNA, thereby suppressing viral replication by reducing viral RNA accumulation. In vitro polymerase assays further elucidated the mechanism of action, demonstrating that DTrip-22 inhibits the poly (U) elongation activity of EV-A71 3Dpol without affecting VPg uridylation activity [125]. However, the substitution of lysine for Arg 163 in EV-A71 3Dpol can cause drug resistance in the virus. GPC-N114 is an inhibitor with broad-spectrum anti-enterovirus and cardioviral activity that inhibits EV-A71 with an EC_50_ value of 0.13 μM. The resolution of the crystal structure of an inhibitor of GPC-N114 binding to CV-B3 3Dpol confirmed the RNA-binding channel as a target of GPC-N114 [126]. BPR-3P0128 is a highly potent antiviral drug against EV-A71 with an EC_50_ value of 0.0029 μM [126]. It inhibits EV-A71 RNA-dependent RNA polymerase activity and VPg uridylation synthesis in vitro and is also active against DTrip-22-resistant EV-A71 viruses carrying the 3D-R163 K mutant [127]. Aurintricarboxylic acid is also a non-nucleoside analog found to inhibit EV-A71 RNA synthesis by inhibiting in vitro 3D RdRp activity [128].

Viral polymerases represent a highly sought-after target for antiviral drugs. The advantages of most nucleoside or nucleotide analogs targeting viral 3D pol include high potency, a high genetic barrier to resistance, and broad-spectrum antiviral activity—most function by mimicking normal nucleosides or nucleotides to participate in viral RNA synthesis. Once incorporated into the growing RNA chain, they cause chain termination, directly inhibiting viral RNA synthesis. Non-nucleoside inhibitors act through distinct mechanisms and therefore may show reduced cross-resistance with nucleoside analogs. One of the major challenges in developing nucleoside or nucleotide polymerase inhibitors is potential off-target effects on host polymerases, particularly mitochondrial polymerases. Overall, viral 3D polymerase represents a promising target for antiviral drug development.

**Figure 8:**
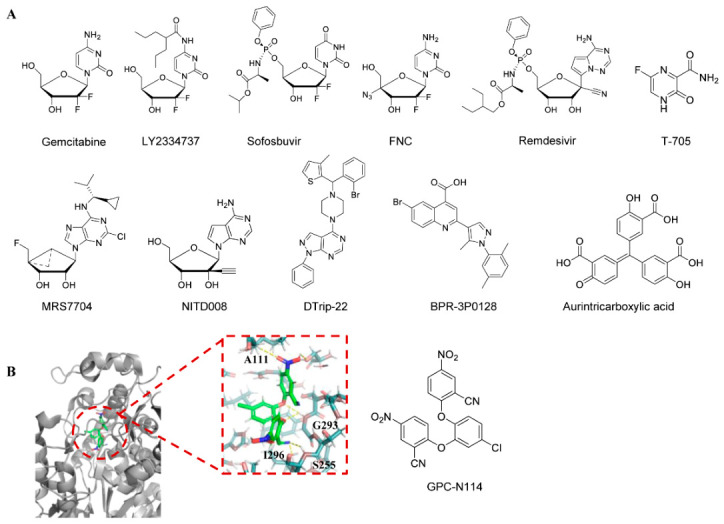
**In panel (**B**), nitrogen and oxygen atoms are shown in blue and red, respectively, and the inhibitor carbon atoms are shown in green.** (**A**) Chemical structure of inhibitors targeting 3D proteins in EVs. (**B**) Crystal structure of CV-B3-3D polymerase in complex with GPC-N114 inhibitor (PDB:4Y2A) and the chemical structure of GPC-N114.

Figure 8 shows the structural formulas of all small-molecule compounds that bind to the 3D protein, as well as the interaction model between GPC-N114 and the 3D protein.

### 2.8. Viral Release Inhibitors

This strategy targets intracellular vesicle transport pathways. Retro-2^cycl^ and Retro-2.1 are inhibitors of several pathogens that specifically target intracellular vesicle transport, which are also involved in EV-A71 life cycle processes, including progeny virus release [129]. In the cytopathic effect inhibition assay, the half-effective concentrations of Retro-2^cycl^ and Retro-2.1 were 12.56 μM and 0.05 μM, respectively, and both inhibited EV-A71 infection with low cytotoxicity. Furthermore, Retro-2cycl administration at a dose of 10 mg/kg significantly protected 90% of newborn mice from lethal EV-A71 challenge.

**Figure 9:**
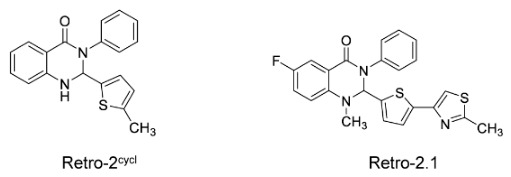
Viral release inhibitors: Chemical structure of Retro-2cycl and Retro-2.1.

Figure 9 shows the chemical structures of Retro-2^cycl^ and Retro-2.1.

### 2.9. Internal Ribosome Entry Site (IRES) Inhibitors

Enterovirus IRES is roughly 450 nucleotides long and is found in the 5′ non-coding region (5′ UTR) of the viral genome. It is a highly organized RNA sequence with conserved nucleotide sequence modules and several stem-loop structures [130]. The intricate three-dimensional spatial structure created by the interactions of these stem-loop structures serves as a crucial foundation for IRES’s operations [131]. The enterovirus IRES has the ability to directly engage ribosomes, allowing viral mRNA translation to begin without a cap structure, in contrast to the majority of eukaryotic mRNAs that depend on a 5′-end cap structure to recruit ribosomes for translation commencement. This indicates that it might be possible to produce antiviral effects by specifically interfering with IRES-mediated viral translation.

Emetine is an antiprotozoal drug that was subsequently found to exhibit activity against a range of human enteroviruses at nanomolar concentrations, including CV-A16, CV-B1, EV-D68, and ECHO-6 [132]. Among them, the drug had an obvious inhibitory effect on EV-A71, with an EC_50_ value of 0.04 μM and a CC_50_ value of 10 μM. Notably, animals administered with emetine demonstrated 100% survival protection at doses as low as 0.2 mg/kg twice daily when evaluated in vivo in an EV-A71-infected mouse model. Furthermore, compared to the solvent-treated control group, the emetine group exhibited considerably reduced viral loads in a number of infected mice’s organs, including the brain, spleen, and fore and hindlimbs. The potential importance of emetine in EVs treatment research is highlighted by this finding.

Idarubicin (IDR) is a topoisomerase II inhibitor and an anthracycline approved by the FDA for the treatment of tumors [133]. The drug has now been identified as a broad-spectrum antiviral drug against enteroviruses. Studies have shown that IDR inhibits EV-A71 IRES-mediated viral protein translation but not host P53 IRES activity, suggesting that IDR may be selective for viral IRES [134]. In addition, IDR blocks the binding of EV-A71 IRES to the host IRES trans-acting hnRNPA 1.

Currently, multiple heparan sulfate (HS) mimetics have been developed for EVs, including heparin, heparan sulfate, and pentosan polysulfate. Among these, heparin has been identified as the most potent inhibitor, suppressing viral replication by over 90% at a concentration of 7.81 μg/mL. Mechanistic studies indicate that heparin inhibits early stages of viral replication by blocking viral attachment to cells [135].

To identify potential therapeutic agents for HFMD, Saravanan Gunaseelan and colleagues screened a flavonoid library of 502 compounds. Through cellular permeability and viral plaque assays, they identified prunin as the most potent inhibitor of EV-A71 with an EC_50_ of 115.3 nM. In BALB/c mice infected in vivo, prunin effectively reduced EV-A71-associated clinical symptoms and mortality. However, it is important to note that prunin is a narrow-spectrum antiviral agent that is effective against enteroviruses A and B but not against enterovirus C, rhinovirus A, and other viruses [136]. In a similar vein, licochalcone A was identified as an antiviral compound against enteroviruses through neutralization screening assays [137]. Chuang et al. discovered that licochalcone A significantly suppressed EV-D68 replication by inhibiting viral IRES-dependent translation. It also exhibited inhibitory activity against CV-B3 and EV-A71. Licochalcone A is a characteristic chalcone isolated from the roots of liquorice (*Glycyrrhiza* species), widely used in traditional Chinese medicine.

Flavonoids are a large class of natural compounds with various biological activities. Several flavonoids have been demonstrated to have inhibitory effects on EV-A71 in cell cultures [138]. For example, apigenin, luteolin, kaempferol, and formononetin provided survival protection rates of 88.89%, 91.67%, 88.89%, and 75%, respectively, against the lethal attack of EV-A71 [138]. It is notable that isorhamnetin provided 100% maximum mouse survival protection at a dose of 10 mg/kg. This indicates that flavonoids have great potential in future drug research for anti-EVs.

The stem-loop II structure at the IRES of EV-A71 is critical for viral replication and represents a novel drug target. Screening of a focused library of RNA-targeting compounds using peptide exchange assays revealed DMA-135 as an effective IRES inhibitor. DMA-135 inhibits EV-A71 replication in cells with an IC_50_ value of 7.54 μM [139]. It is proposed that upon binding to DMA-135, SLII undergoes a conformational change that stabilizes the ternary complex with the AUF1 protein, thereby inhibiting translation.

All things considered, IRES is still a novel target with much room for inhibitor design. Combination therapy may benefit greatly from the natural nature of the majority of IRES-targeting inhibitors, some of which are derived from traditional Chinese herbal medications.

**Figure 10:**
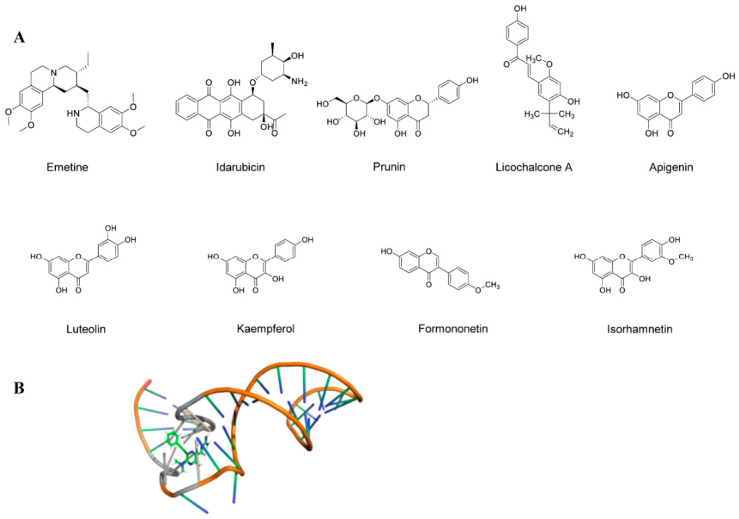
(**A**) The chemical structure of IRES inhibitors. (**B**) Solution NMR structure of EV-A71 IRES in complex with DMA-135 (PDB:6XB7). DMA-135 is shown in green, the EV-A71 IRES RNA backbone is shown in orange, and the local RNA nucleotides around the binding site are shown in blue/gray.

Figure 10 shows the chemical structures of all small-molecule compounds targeting the IRES, as well as the solution NMR structure of the EV-A71 IRES–DMA-135 complex.

## 3. Host Proteins Involved in Virus Replication

In antiviral therapy, besides drugs that directly target the virus itself, there is another class of drugs that exert antiviral effects by acting on host cells. These drugs exploit the virus’s dependence on host cells, interfering with the virus’s life cycle within the host cell to inhibit viral replication and spread, thereby providing new strategies for antiviral treatment. Maraviroc is a typical example among them. Maraviroc is a CCR5 antagonist, primarily used to treat HIV infection [140]. It specifically binds to the CCR5 receptor, altering its conformation to block the interaction between HIV-1 envelope glycoprotein gp120 and CCR5, thereby inhibiting viral entry into host cells. Compounds of this type are also found in the research of anti-EVs drugs.

### 3.1. eIF4A

Among the various host factors exploited by enteroviruses, eukaryotic initiation factor 4A (eIF4A) is an ATP-dependent RNA helicase that plays a crucial role in viral protein synthesis. It facilitates the unwinding of the highly structured internal ribosomal entry site (IRES) element within the viral 5′ untranslated region. At the molecular level, silvestrol binds to eIF4A and stabilizes its interaction with RNA, effectively clamping the helicase onto its RNA substrate [141,142]. This abnormal stabilization prevents eIF4A recycling and leads to functional depletion of active eIF4A from the eIF4F complex, resulting in a profound inhibition of cap-dependent translation initiation. Rocaglamide A (Roc-A) is a natural compound derived from plants of the genus Aglaia. It exhibits significant, dose-dependent inhibitory effects against EV-A71 (with concentrations of 10–100 nM markedly reducing viral titers) and, in animal studies, significantly improves survival rates and delays the onset of neurological symptoms [143].

### 3.2. AP2M1

Recent studies have identified a conserved host–virus interaction that may serve as a potential target for the development of broad-spectrum antiviral agents. The interaction between the host adaptor protein complex 2 subunit mu 1 (AP2M1) and the YxxΦ motif present in viral proteins is critical for the intracellular trafficking of several viruses. In enteroviruses, including EV-A71 and EV-D68, a conserved YxxΦ motif has been identified in the viral 2C protein [144]. AP2M1 facilitates the localization of the EV-A71 2C protein to endoplasmic reticulum (ER)-associated membranes, which is important for viral replication. A chemical screening study identified N-(p-amylcinnamoyl)anthranilic acid (ACA) as an inhibitor of the AP2M1–YxxΦ interaction without affecting AP2M1 phosphorylation [76]. ACA treatment reduced the colocalization between the 2C protein and the ER and exhibited antiviral activity against EV-A71. Moreover, ACA has been reported to display broad-spectrum antiviral activity against several viruses, including the influenza virus, Zika virus, and MERS-CoV, both in vitro and in vivo.

### 3.3. Host Proteins Associated with 3A Proteins

In the 3A pro inhibitors module of this paper, compounds targeting PI4KB inhibitors and OSBP inhibitors have been discussed. Additionally, GW4869 indirectly suppresses enterovirus replication and transmission by interfering with the production and release of host cell exosomes/extracellular vesicles. By inhibiting nSMase, it reduces intracellular ceramide levels, thereby suppressing exosome generation via an ESCRT-independent pathway [145].

### 3.4. HSP90

Heat shock protein 90 (HSP90) is an essential host molecular chaperone that regulates the folding, stabilization, and functional maturation of a wide range of client proteins, including multiple viral non-structural proteins required for efficient replication [146]. Increasing evidence indicates that enteroviruses exploit the HSP90 chaperone machinery to ensure the correct conformational maturation and stability of key components of the viral replication complex. Pharmacological inhibition of HSP90 using small-molecule inhibitors such as geldanamycin and its less toxic derivative 17-allylamino-17-demethoxygeldanamycin (17-AAG) has been shown to markedly suppress enterovirus replication in vitro [147]. These compounds bind to the ATP-binding pocket of HSP90, thereby disrupting its chaperone activity and promoting proteasomal degradation of immature or misfolded viral proteins [143]. As a result, viral polyprotein processing, replication complex assembly, and RNA synthesis are significantly impaired. Targeting HSP90 thus represents a promising host-directed antiviral strategy that offers broad-spectrum activity and a high genetic barrier to resistance. However, concerns regarding cytotoxicity and the therapeutic window remain important considerations for clinical translation.

### 3.5. DHODH

Through phenotypic screening as well as subsequent SAR studies, the small-molecule compound RYL-634 has been identified as having excellent broad-spectrum inhibitory activity, which may be useful for the development of novel therapeutic agents. It inhibits multiple viruses, including the hepatitis C virus, dengue virus, Zika virus, EV-A71, human immunodeficiency virus, and respiratory syncytial virus, and additional viruses can be inhibited [148]. Among these, RYL-634 had an EC_50_ value of 4 nM for EV-A71. To identify the target of RYL-634, the researchers designed and synthesized a probe tagged with a cross-linker and an alkyne. The probe was then incubated with live cells in vivo or in lysis in vitro to bind to target proteins, thereby establishing that the target of RYL-634 was dihydroorotate dehydrogenase (DHODH). During the validation using RYL-634, researchers observed no mutant resistance and identified potent synergistic effects with several FDA-approved drugs, demonstrating significant potential for developing new broad-spectrum antivirals based on RYL-634. In addition, the DHODH inhibitor ML390 was found to have potential anti-EV-A71 activity, with IC_50_ and selectivity index values of 0.06601 μM and 156.5, respectively [149]. Brequinar is effective in inhibiting multiple viral replication; however, its antiviral activity can be reversed by supplementation with exogenous pyrimidines, indicating that the antiviral effect of brequinar against enteric viruses is dependent on inhibition of DHODH activity [150]. These findings collectively demonstrate that DHODH is not only a critical host factor for viral replication but also that structurally diverse inhibitors of DHODH represent potent antiviral agents against both RNA and DNA viruses [151].

### 3.6. ER

Host cellular signaling pathways are extensively exploited by enteroviruses to facilitate viral entry, genome replication, protein synthesis, and virion assembly. Among these pathways, estrogen receptor (ER) signaling and the mitogen-activated protein kinase/extracellular signal-regulated kinase (MAPK/ERK) cascade have been implicated in creating intracellular environments permissive for viral replication. The selective estrogen receptor modulator tamoxifen has been reported to inhibit enteroviral replication in cell culture systems, possibly through the modulation of ER signaling, lipid metabolism, and endosomal trafficking. In parallel, small-molecule inhibitors targeting the MAPK/ERK pathway, including the MEK inhibitors U0126 and PD98059, as well as the multi-kinase inhibitor regorafenib, suppress viral replication by interfering with ERK activation and the downstream transcriptional programs required for efficient viral RNA synthesis [152,153,154].

### 3.7. Endolysosomal Pathway as a Host-Targeting Antiviral Strategy

In addition to the host factors discussed above, the endolysosomal pathway has emerged as an important host-directed antiviral target for enteroviruses. Enteroviruses typically enter host cells via endocytosis and rely on endosomal acidification and membrane trafficking for viral uncoating and genome release. Therefore, disruption of endolysosomal trafficking and maturation represents a promising antiviral strategy.

Niclosamide is an important broad-spectrum antiviral drug that is effective against enteroviruses. Niclosamide inhibits viral infection at an early stage by neutralizing endosomal acidification, thereby blocking virus entry. Mechanistically, niclosamide acts as a proton carrier that disrupts endosomal pH homeostasis. Meanwhile, researchers also found that combining niclosamide with bafilomycin A1 increased antiviral efficacy by nearly 60 times compared to using either drug alone. This demonstrates that the drugs exhibit synergistic antiviral effects, which is of great importance for drug development [155].

Among host-directed antiviral drugs targeting the endoplasmic reticulum-lysosome pathway, sertraline is a drug of particular interest. Sertraline accumulates in acidic compartments such as late endosomes and lysosomes, where it neutralizes the endolysosomal pH and inhibits EV-A71 infection [156].

**Figure 11:**
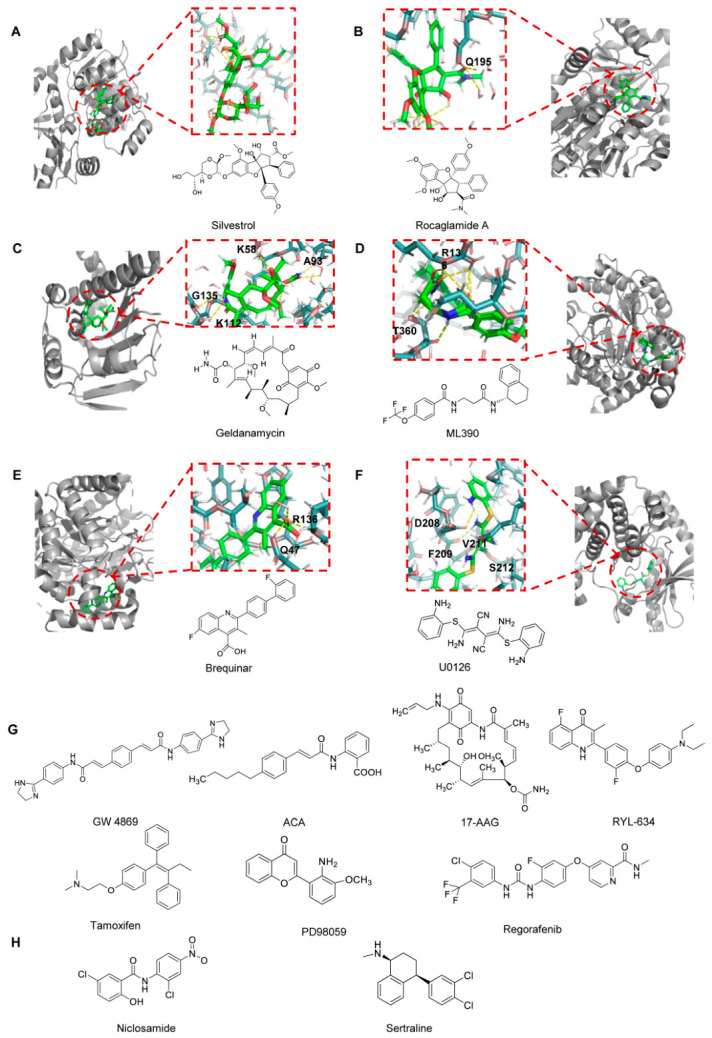
**In panels (**A**–**F**), the ligand is shown in green, with nitrogen and oxygen atoms shown in blue and red, respectively.** (**A**) Crystal structure of the human eIF4A-1 in complex with silvestrol (PDB:9AVR) and chemical structure of silvestrol. (**B**) Crystal structures of the human eIF4A-1 in complex with rocaglamide A (PDB:5ZC9) and chemical structure of rocaglamide A. (**C**) Crystal structures of the HSP90 in complex with geldanamycin (PDB:1YET) and chemical structure of geldanamycin. (**D**) Crystal structures of the DHODH in complex with ML390 (PDB:5K9C) and chemical structure of ML390. (**E**) Crystal structures of the DHODH in complex with brequinar (PDB:1D3G) and chemical structure of brequinar. (**F**) Crystal structures of MEK1 in complex with U0126 (PDB:3EQH) and chemical structure of U0126. (**G**) Chemical structure of the host-targeting antivirals inhibitor for EVs. (**H**) Chemical structure of the endolysosomal pathway antivirals inhibitor for EVs.

Figure 11 primarily shows the structural formulas of all small-molecule compounds that interact with the host protein, as well as interaction models for some of these compounds with the protein.

## 4. Discussion

This review provides a systematic overview of antiviral drug research and development against enteroviruses, focusing on small-molecule inhibitors with clear mechanisms of action or proven efficacy in vivo. Enterovirus infections, especially those caused by EV-A71, CV-A16, and EV-D68, have grown to be major global public health problems because they can result in serious clinical consequences like neurological abnormalities and hand-foot-and-mouth disease. Nevertheless, there are presently no FDA-approved antiviral medications on the market that particularly target these infections. Although several vaccines are already on the market, they also have certain limitations. While VP1me may address this issue, its true application requires substantial follow-up research to validate its potential, as it has only demonstrated immunogenicity but not protective efficacy.

Research indicates that multiple potential inhibitors have been identified as targeting various stages of the viral life cycle. Capsid inhibitors (e.g., pleconaril, vapendavir) bind to the VP1 protein to block viral attachment or uncoating; protease inhibitors (such as the 3C pro-targeting rupintrivir) block viral replication by inhibiting the processing of viral polyproteins and cleavage of host proteins; RNA-dependent RNA polymerase (3Dpol) inhibitors, such as remdesivir and favipiravir nucleoside analogs, directly inhibit viral RNA synthesis. In addition, host-targeting strategies targeting host factors essential for viral replication (such as PI4KB, OSBP), and translation inhibitors targeting viral Internal ribosome entry site (IRES), such as isorhamnetin, also provide new directions for the development of broad-spectrum antiviral drugs.

The field of enterovirus antiviral drug development currently exhibits core characteristics while also facing challenges and future opportunities. First is its broad-spectrum nature and resistance characteristics. Enteroviruses exhibit numerous serotypes and high mutation rates, making single-target drugs highly susceptible to resistance due to viral mutations. To address this challenge, drug combination strategy shows great potential. Research indicates that the combination of drugs with different mechanisms of action can not only produce synergistic effects and improve efficacy, but also significantly improve the genetic barrier for drug resistance of viruses. This is undoubtedly an important direction of clinical treatment in the future. Secondly, the trade-off between virus-targeting and host-targeting strategies should also be taken into account. Virus-targeted drugs (such as protease and polymerase inhibitors) usually have high efficacy and rapid onset, but face the risk of drug resistance. Host-targeted drugs (such as PI4KB inhibitors, OSBP antagonists) theoretically have higher broad-spectrum and lower risk of resistance because host factors are less prone to mutation. However, their primary challenge lies in potential cytotoxicity and off-target effects, which may disrupt normal cellular physiology and cause adverse reactions. Beyond traditional small molecules, peptide-based drugs (e.g., SP40) block viral infection by mimicking receptors or stabilizing capsids, offering high specificity and low susceptibility to resistance. Despite challenges like poor in vivo stability and delivery difficulties, advances in peptide modification techniques (e.g., cyclization, pegylation) and nano-delivery [157] systems position these drugs for significant roles in local or systemic therapies. Simultaneously, screening lead compounds from natural products (e.g., magnolol, flavonoids) represents a fruitful direction. These compounds often have the characteristics of multi-target effects and a good safety profile, which provide a rich chemical library for the new use of old drugs and new drug design.

Although many drugs cannot be commercialized, the reasons behind this are still worth considering. First, toxicity and safety concerns remain major obstacles, particularly for host-targeting agents. Second, rapid viral mutation and drug resistance present significant challenges. Enteroviruses exhibit high mutation rates, which can quickly lead to resistance against single-target antivirals, especially capsid inhibitors and protease inhibitors. Third, suboptimal pharmacokinetic properties have limited clinical development. For example, rupintrivir demonstrated potent antiviral activity in vitro but showed poor oral bioavailability and limited clinical efficacy, ultimately leading to the discontinuation of its development. Fourth, a narrow antiviral spectrum is another important limitation. Finally, a lack of appropriate animal models and clinical trial challenges also contributes to the translational gap. Enterovirus infections primarily affect children, making clinical trials more complex, thus increasing regulatory barriers.

Importantly, the preclinical evidence summarized here is still dominated by neonatal or immunocompromised mouse models, whereas key experimental variables such as sex and group size are not always consistently reported in the original studies. To enable readers to visualize the experimental animal models more clearly and intuitively, Table 2 summarizes, for all compounds in this manuscript with reported animal data, the available information on animal species/strain, age, sex, group size, infection route or model-establishment method, and drug administration regimen. When a field was not explicitly stated in the source article, it was marked as NR rather than inferred.

Among the studies already cited in this review, only a limited subset used human-derived systems. The clearest example is the pleconaril/AG7404/mindeudesivir combination, which showed synergistic antiviral activity in human cell and organoid cultures, and was additionally evaluated in pancreatic β-cell cultures and infected cardiac organ tissues [103]. By contrast, true human clinical evidence remains scarce; notably, the fluoxetine study cited in this review did not demonstrate convincing efficacy in patients with EV-D68-associated AFM [64]. These examples, summarized in Table 3, highlight the need to prioritize organoids, primary human cells, iPSC-derived tissues, and other human-relevant systems in future enterovirus antiviral research.

In summary, although the path to developing drugs targeting EVs is fraught with challenges, researchers are poised to overcome this hurdle in the future through a multi-pronged, multidisciplinary approach. This includes advancing combined therapies and deepening exploration of host-targeted treatments, thereby providing effective therapeutic tools to address the public health threats posed by EVs.

## 5. Conclusions

Enteroviruses remain a significant global health threat, particularly in children, and effective antiviral therapies for severe infections are still unavailable. This review highlights recent progress in enterovirus antiviral development, encompassing inhibitors targeting viral capsid proteins, proteases, replication-associated proteins, RNA-dependent RNA polymerase, and IRES elements, as well as host-directed strategies. Although many candidates demonstrate potent in vitro and in vivo activity, clinical translation has been limited by issues that include toxicity, pharmacokinetics, and resistance. Future antiviral development should prioritize structure-guided optimization, improved drug-like properties, and rational combination therapies targeting both viral and host factors. Advancing our understanding of virus–host interactions will be essential for the development of safe, broad-spectrum, and clinically effective enterovirus antivirals. Antiviral combinations are particularly attractive because they can concurrently target different stages of the viral life cycle, improve therapeutic efficacy through synergistic or additive effects, and increase the genetic barrier to resistance compared with monotherapy.

Moreover, the development of novel anti-enteroviral compounds is also essential for preparedness against future poliomyelitis outbreaks, as such agents may complement vaccination by reducing viral replication and shedding, limiting transmission, and providing additional treatment options in the setting of severe disease or vaccine-derived poliovirus emergence.

A comprehensive summary of the text is presented in Table 4 and Table 5.

## Figures and Tables

**Table 1:** Classification of human enteroviruses.

Types of Enteroviruses	Pathogen
Human enterovirus group A	EV71, EV76, EV89–92, EV114, EV119–121, CA2–8, CA10, CA12, CA14, CA16
Human enterovirus group B	CA9, CVB1–6, EV69, EV73–75, EV77–88, EV93, EV97–101, EV106, EV107, EV110–113
Human enterovirus group C	CA1, CA11, CA13, CA17, CA19–22, CA24, EV95, EV 96, EV99, EV102, EV104, EV105
Human enterovirus group D	EVD68, EVD70
Human Rhinovirus Group A	RV-A1, 2, 7–13, 15, 16, 18–25, 28–34, 36, 38–41, 43, 45–47, 49–51, 53–68, 73–78, 80–82, 100–109
Human Rhinovirus Group B	RV-B6, 14, 17, 26, 27, 35, 37, 42, 48, 52, 69, 70, 72, 79, 83, 84, 86, 91–93, 97, 99, 100–106
Human Rhinovirus Group C	RV-C1-55

**Table 2:** Summary of animal models used for antiviral candidates discussed in this manuscript.

Compound	Virus/Model	Animal Model (Species/Strain; Age; Sex; *n*)	How the Model was Established	Drug Administration	Reference
11526092	EV-D68 respiratory model	AG129 mice; 4 weeks; male and female; total *n* = 65 randomized into five groups of 12 plus one normal group of 6	Intranasal inoculation of mouse-passaged EV-D68 MP30 PP (1 × 10^4.5^ CCID_50_ in 90 μL MEM) after ketamine anesthesia	Oral gavage once daily for 5 days, starting 2 h before infection	[39]
11526092	CV-B5 systemic model	BALB/c mice; 4–5 weeks; male; *n* = 6/group; intact control *n* = 10	Intraperitoneal inoculation of mouse-adapted CV-B5 (6 × 10^6^ TCID_50_/0.2 mL)	Intraperitoneal dosing 100 mg/kg, twice daily, from 0.5 day before infection to day 5 p.i.	[39]
Schizonepeta tenuifolia extract (STE)	EV-A71 MP4 lethal model	ICR mice; 7 days old; sex NR; *n* = 16 STE, *n* = 15	Intraperitoneal inoculation of 2 × 10^6^ PFU EV-A71 MP4 in 10 μL DMEM	Intraperitoneal STE 250 mg/kg once daily for 14 days, starting 1 day after infection	[50]
Telaprevir	EV-D68 acute flaccid myelitis model	Swiss Webster mouse pups; 2 days old; sex NR; *n* NR in accessible source	EV-D68 challenge at 1000 TCID_50_ in a murine AFM model; full route/group size not retrievable from accessible full text	Telaprevir 35 mg/kg; prophylactic dosing reduced paralysis; route not fully retrievable from accessible full text	[54]
2C-6I (dibucaine derivative)	EV-A71 mouse model	Mice; age/strain/sex/*n* NR in accessible source		Compound 2C-6I showed in vivo efficacy and synergy with emetine; full dosing schedule NR in accessible source	[73]
Jun6504	EV-D68 paralytic model	Swiss Webster mice; postnatal day 2; equal males and females; *n* NR in accessible lines	Intramuscular injection into left quadriceps with 10 μL EV-D68 (10,000 × TCID_50_/pup)	Intraperitoneal 50 mg/kg daily, starting within 30 min or 24 h after infection	[75]
Peptide 2CL	EV-A71 lethal challenge	ICR mice; 1 day old; sex NR; *n* = 9 treated, *n* = 10 vehicle, *n* = 8 mock	Intraperitoneal challenge with 10^7^ PFU EV-A71 strain H (VR-1432)	Intraperitoneal 20 mg/kg at 1 h p.i., then twice daily for 7 consecutive days	[77]
CAA triple combination (pleconaril + MDL-860 + oxoglaucine)	CV-B3 mouse infection model	Mice; strain/age/sex/*n* NR in accessible abstract		Consecutive alternating administration: one drug/day in a repeating 3-day cycle for a total of 12 days	[154]
Vemurafenib	Acute enterovirus mouse model/CV-B4 tissues	Mice; strain/age/sex/*n* NR in accessible abstract	Acute mouse model reported; accessible source states reduced virus in pancreas and heart	Dose/route not stated in accessible abstract of the cited article	[86]
G197 + CUR-N373	EV-A71 lethal challenge	BALB/c neonates; 6 days old; sex NR; *n* not explicitly stated in accessible lines	Lethal EV-A71 challenge with a single 2 × 10^7^ PFU dose	Intraperitoneal dosing at 1 mg/kg each; one dose 2 h before infection plus daily dosing for 6 additional days	[41,89]
FNC	EV-A71 and CV-A16 neonatal models	Neonatal mice; strain/sex/n NR in accessible source	Neonatal mouse challenge models reported for EV-A71 and CV-A16	1 mg/kg every 2 days protected against mortality and reduced tissue viral loads	[117]
NITD008	EV-A71 systemic model	AG129 mice; 2 weeks old; sex NR; *n* = 8–10/group	Intraperitoneal inoculation with 10^7^ PFU EV71 strain S41 per mouse	Oral 5 mg/kg twice daily from the day of infection for 3 consecutive days	[123]
Retro-2cycl	EV-A71 lethal challenge	Newborn mice; strain/sex/n NR in accessible source		10 mg/kg significantly protected 90% of newborn mice	[128]
Emetine	EV-A71 mouse model	KM mice (reported in accessible source); age/sex/n NR in accessible source	EV-A71 infection model	Oral 0.20 mg/kg twice daily; complete protection reported	[131]
Prunin	EV-A71 model	BALB/c mice; age/sex/*n* NR in accessible source	EV-A71 strain 41 lethal challenge model reported	3 mg/kg and 10 mg/kg achieved 100% survival in cited secondary summary; exact route/schedule not retrievable from accessible primary source	[135]
Apigenin/luteolin/kaempferol/formononetin/isorhamnetin	EV-A71 lethal challenge	BALB/c mice; newborn (<24 h); sex NR; *n* = 15/group	Intracranial inoculation with 600,000 TCID_50_ WT-EV-A71 within 24 h of birth	Intraperitoneal injections in 10% DMSO-PBS at tested doses for 7 consecutive days	[137]
Rocaglamide A (Roc-A)	EV-A71 neuropathogenesis model	Mice; strain/age/sex/*n* NR in accessible source	EV-A71-infected mouse neuropathogenesis model	Roc-A treatment prolonged survival and lowered virus loads in spinal cord and brain; dose/route not fully retrievable from accessible source	[142]
GW4869	EV-A71 pathogenesis model	Mice; likely suckling model; exact strain/age/sex/*n* NR in accessible abstract	EV-A71-infected mouse model used to assess extracellular-vesicle biogenesis blockade	GW4869 reduced viral load/pathogenesis in multiple tissues; detailed dose/route not stated in accessible abstract	[144]

**Table 3:** Human-derived, organoid, and human evidence among the studies cited in this review.

Compound/Regimen	Model Type	Main Finding	Reference
Pleconaril + AG7404 + mindeudesivir	Human cell and organoid cultures; pancreatic β-cell cultures; infected human cardiac organ tissues	Synergistically inhibited enterovirus replication, did not disturb glucose/insulin levels in β-cell cultures, and preserved the contraction rhythm of infected cardiac organ tissues	[103]
Fluoxetine	Human clinical data (AFM patients)	Cited human study did not show convincing efficacy in EV-D68-associated AFM, highlighting the translational gap between preclinical and patient studies	[64]
Vemurafenib	Human-derived intestinal epithelial cells and pancreatic β cells	Replication of diabetogenic enteroviruses was inhibited in human-derived epithelial and β-cell systems	[87]

**Table 4:** Summary of antiviral compounds targeting enteroviruses and their mechanisms of action.

Target Protein/Pathway	Compound(s)	Mechanism of Action	Reference
Capsid (VP1)	Pleconaril	Binds VP1 hydrophobic pocket, blocks uncoating and RNA release	[29,30]
	Vapendavir	Capsid-binding inhibitor (VP1)	[31,32]
	Pocapavir	Capsid inhibitor (VP1 binding)	[31]
	Pirodavir/BTA-798	Capsid-binding inhibitor	[33,35]
	Tanomastat	Prevents capsid dissociation via VP1 binding	[37]
	PR66, NLD, ALD	Bind VP1, inhibit viral entry/uncoating	[38]
	11526092	Binds VP1 hydrophobic pocket (cryo-EM confirmed)	[39]
	R856932	Binds VP1 pocket, blocks uncoating	[40]
	G197	Capsid-binding inhibitor (VP1)	[41]
Capsid (VP3)	PF4/C15 peptide	Binds VP3 or receptor SCARB2, blocks attachment	[45,46]
2A protease	LVLQTM peptide	Inhibits 2Apro cleavage activity	[49]
	CW-33	Inhibits IFNAR1 cleavage, restores IFN signaling	[52]
	Telaprevir	Inhibits 2Apro (irreversible biphasic mechanism)	[54,55]
	Jun11762	Optimized 2Apro inhibitor (structure-based)	[56]
2B protein	DIDS	Blocks 2B ion channel activity	[57]
	CD74	Interacts with 2B, inhibits replication	[58]
2C protein	Guanidine hydrochloride	Inhibits 2C-mediated replication	[62,67,68]
	Fluoxetine	Inhibits viral RNA/protein accumulation (2C target)	[62]
	2C-12b	Inhibits 2C function (broad-spectrum)	[66]
	HBB/TBZE-029	Bind ATP-binding pocket, inhibit ATPase	[69,70]
	Dibucaine and analogs	Target 2C (mechanism via resistance mapping)	[67,71]
	JX040/Jun571/Jun6504	Inhibit 2C protein activity	[74,75]
	2CL peptide	Disrupts 2C oligomerization	[77]
3A–PI4KB pathway	Enviroxime	Inhibits PI4KB recruitment	[81]
	GW5074/AN-12-H5	Enviroxime-like (PI4KB pathway)	[82,83]
	MDL-860	Covalent PI4KB inhibitor (allosteric site)	[85]
	Vemurafenib	Targets PI4KB pathway	[87,88]
	CUR-N373/CUR-N399	PI4KB inhibitors	[89,90]
OSBP pathway	OSW-1	OSBP antagonist	[91,92]
	Itraconazole	Inhibits OSBP	[93,94]
	TTP-8307	Blocks PI4P/cholesterol transport (OSBP)	[83]
3C protease	Rupintrivir	Inhibits 3Cpro protease activity	[99]
	AG7404	3Cpro inhibitor	[102,103]
	NK-1.8k/SG85/Aldehyde 5x	Peptidomimetic 3Cpro inhibitors	[105,106,107,108]
	GC373/GC376	3Cpro inhibitors	[113]
	Quercetin	Binds 3Cpro substrate pocket	[115]
3D polymerase	Remdesivir	Nucleoside analog, inhibits RNA synthesis	[121]
	Favipiravir	Targets 3Dpol	[122,123]
	FNC	Competitive inhibitor of 3Dpol	[118]
	NITD008	Inhibits RNA synthesis	[124]
	DTrip-22	Inhibits RNA accumulation	[125]
	GPC-N114	Targets RNA-binding channel of 3Dpol	[126]
IRES	Emetine	Inhibits IRES-mediated translation	[132]
	Idarubicin	Blocks IRES–hnRNPA1 interaction	[134]
	DMA-135	Binds SLII, inhibits translation	[139]
Host factors	Silvestrol/Roc-A	Target eIF4A, inhibit translation	[141,142,143]
	ACA	Blocks AP2M1–viral interaction	[76]
	Geldanamycin/17-AAG	HSP90 inhibitors	[147]
	RYL-634/ML390/Brequinar	DHODH inhibitors	[148,149,150]

**Table 5:** Antiviral combination strategies against enteroviruses (with experimental models).

Combination	Model	Outcome	Reference
Pleconaril + Rupintrivir + Remdesivir	A549 cells	Enhanced efficacy vs. mono/dual therapy	[34]
V-073 + BTA-798	In vitro	Synergistic antiviral effect	[36]
CW-33 + IFN-β	Cell-based	Synergistic antiviral effect	[52]
Pleconaril + AG7404 + Mindeudesivir	Organoid/cell	Broad-spectrum inhibition	[103]
CUR-N373 + G197	Mouse	Improved survival, reduced pathology	[41]
Remdesivir + Favipiravir	Cell		[122]
2C-6I + Emetine	Mouse	Synergistic effect	[73]
CAA (Pleconaril + MDL-860 + Oxoglaucine)	In vivo	Increased survival, reduced resistance	[154]
Niclosamide + Bafilomycin A1	Human cell infection model (RD, HeLa, Vero)	~60-fold enhanced antiviral efficacy; synergistic effect	[155]

## Data Availability

No new data were created in this study. Data sharing is not applicable to this article.

## References

[1] Zell R., Delwart E., Gorbalenya A.E., Hovi T., King A.M.Q., Knowles N.J., Lindberg A.M., Pallansch M.A., Palmenberg A.C., Reuter G. (2017). ICTV Virus Taxonomy Profile: *Picornaviridae*. J. Gen. Virol..

[2] Chang Y.K., Chen K.H., Chen K.T. (2018). Hand, foot and mouth disease and herpangina caused by enterovirus A71 infections: A review of enterovirus A71 molecular epidemiology, pathogenesis, and current vaccine development. Rev. Inst. Med. Trop. Sao Paulo.

[3] Tapparel C., Siegrist F., Petty T.J., Kaiser L. (2013). Picornavirus and enterovirus diversity with associated human diseases. Infect. Genet. Evol..

[4] Lin J.Y., Shih S.R. (2014). Cell and tissue tropism of enterovirus 71 and other enteroviruses infections. J. Biomed. Sci..

[5] Puenpa J., Wanlapakorn N., Vongpunsawad S., Poovorawan Y. (2019). The History of Enterovirus A71 Outbreaks and Molecular Epidemiology in the Asia-Pacific Region. J. Biomed. Sci..

[6] Garcia-Pedemonte D., Carcereny A., Andrés C., Anton A., Pérez I., Blanco A., Fuentes C., Costafreda M.I., Nadal-Baron P., Galofré B. (2025). Unveiling the enterovirus diversity in Barcelona, Spain (2020–2024) through wastewater and clinical surveillance. Emerg. Microbes Infect..

[7] Xie Z.F., Khamrin P., Maneekarn N., Kumthip K. (2024). Epidemiology of Enterovirus Genotypes in Association with Human Diseases. Viruses.

[8] Kartbayashi D., Kaida A., Hirai Y., Yamamoto S.P., Fujimori R., Okada M., Kubo H., Iritani N. (2019). An Epidemic of Hand, Foot, and Mouth Disease Caused by Coxsackievirus A6 in Osaka City, Japan, in 2017. Jpn. J. Infect. Dis..

[9] Yin D., Wang C., Wang C., Xiao Z., Ji S. (2018). Epidemiology Characteristics of Human Coxsackievirus A16 and Enterovirus 71 Circulating in Linyi, China, from 2009 to 2017. Jpn. J. Infect. Dis..

[10] Li Y., Yang J.M., Liang L., Wang K., Turtle L., Li P., Zhou Y.H., Shen Y.F., Cui P., Zhou C.C. (2025). Clinical characteristics and severity of hand, foot, and mouth disease by virus serotype: A prospective hospital-based cohort study. PLoS Negl. Trop. Dis..

[11] Teoh H.L., Mohammad S.S., Britton P.N., Kandula T., Lorentzos M.S., Booy R., Jones C.A., Rawlinson W., Ramachandran V., Rodriguez M.L. (2016). Clinical Characteristics and Functional Motor Outcomes of Enterovirus 71 Neurological Disease in Children. JAMA Neurol..

[12] Akinnurun O.M., Encalada M.N., Orth J., Petzold M., Böttcher S., Diedrich S., Smitka M., Schröttner P. (2023). Enterovirus A71-associated acute flaccid paralysis in a pediatric patient: A case report. J. Med. Case Rep..

[13] Zhou J., Wang Y.P., Zhu G.Y., Yan M.Z., Li Z.N., Li L., Kuang J.W., Zhang W.Y., Huang C.L., Ren F.L. (2025). Pilaralisib inhibits the replication of enteroviruses by targeting the PI3K/AKT signaling pathway. Virol. J..

[14] Varanese L., Xu L., Peters C.E., Pintilie G., Roberts D.S., Raj S., Liu M.Y., Ooi Y.S., Diep J., Qiao W.J. (2025). MFSD6 is an entry receptor for enterovirus D68. Nature.

[15] Zhou Y.H., Zhou C.C., Wang K., Qiu Q., Cheng Y.B., Li Y., Cui P., Liang L., Li P., Deng X.W. (2023). Diagnostic performance of different specimens in detecting enterovirus A71 in children with hand, foot and mouth disease. Virol. Sin..

[16] Ren J.Y., Zhang Z.F., Xia Y., Zhao D.Q., Li D.Q., Zhang S.J. (2025). Research Progress on the Structure and Function, Immune Escape Mechanism, Antiviral Drug Development Methods, and Clinical Use of SARS-CoV-2 M^pro^. Molecules.

[17] Wang H.Q., Li Y.H. (2019). Recent Progress on Functional Genomics Research of Enterovirus 71. Virol. Sin..

[18] Su Y.S., Hsieh P.Y., Li J.S., Pao Y.H., Chen C.J., Hwang L.H. (2020). The Heat Shock Protein 70 Family of Chaperones Regulates All Phases of the Enterovirus A71 Life Cycle. Front. Microbiol..

[19] Bello A.M., Chaimongkolnukul K., Poomputsa K., Mekvichitsaeng P., Roshorm Y.M. (2024). Immunogenicity and immunodominant linear B-cell epitopes of a new DNA-based tetravalent vaccine against four major enteroviruses causing hand, foot, and mouth disease. Vaccine.

[20] Zhang X.L., Zhang Y.F., Li H., Liu L.D. (2023). Hand-Foot-and-Mouth Disease-Associated Enterovirus and the Development of Multivalent HFMD Vaccines. Int. J. Mol. Sci..

[21] Zhang W.J., Li Q.J., Yi D.R., Zheng R.F., Liu G.H., Liu Q., Guo S.S., Zhao J.Y., Wang J., Ma L. (2024). Novel virulence determinants in VP1 regulate the assembly of enterovirus-A71. J. Virol..

[22] Yamayoshi S., Yamashita Y., Li J.F., Hanagata N., Minowa T., Takemura T., Koike S. (2009). Scavenger receptor B2 is a cellular receptor for enterovirus 71. Nat. Med..

[23] Nishimura Y., Shimojima M., Tano Y., Miyamura T., Wakita T., Shimizu H. (2009). Human P-selectin glycoprotein ligand-1 is a functional receptor for enterovirus 71. Nat. Med..

[24] Yang S.L., Chou Y.T., Wu C.N., Ho M.S. (2011). Annexin II Binds to Capsid Protein VP1 of Enterovirus 71 and Enhances Viral Infectivity. J. Virol..

[25] Tan C.W., Poh C.L., Sam I.C., Chan Y.F. (2013). Enterovirus 71 Uses Cell Surface Heparan Sulfate Glycosaminoglycan as an Attachment Receptor. J. Virol..

[26] Yang B., Chuang H., Yang K.D. (2009). Sialylated glycans as receptor and inhibitor of enterovirus 71 infection to DLD-1 intestinal cells. Virol. J..

[27] Ren X.X., Ma L., Liu Q.W., Li C., Huang Z., Wu L., Xiong S.D., Wang J.H., Wang H.B. (2014). The molecule of DC-SIGN captures enterovirus 71 and confers dendritic cell-mediated viral *trans*-infection. Virol. J..

[28] Lin J.Y., Kung Y.A., Shih S.R. (2019). Antivirals and vaccines for Enterovirus A71. J. Biomed. Sci..

[29] Pevear D.C., Tull T.M., Seipel M.E., Groarke J.M. (1999). Activity of pleconaril against enteroviruses. Antimicrob. Agents Chemother..

[30] Dahl-Jorgensen K. (2024). Virus as the cause of type 1 diabetes. Trends Mol. Med..

[31] Kalam N., Balasubramaniam V. (2024). Emerging Therapeutics in the Fight Against EV-D68: A Review of Current Strategies. Influenza Other Respir. Viruses.

[32] Sun L., Meijer A., Froeyen M., Zhang L.L., Thibaut H.J., Baggen J., George S., Vernachio J., Kuppeveld F.J.M., Leyssen P. (2015). Antiviral Activity of Broad-Spectrum and Enterovirus-Specific Inhibitors against Clinical Isolates of Enterovirus D68. Antimicrob. Agents Chemother..

[33] Rhoden E., Zhang M.Y., Nix W.A., Oberste M.S. (2015). In Vitro Efficacy of Antiviral Compounds against Enterovirus D68. Antimicrob. Agents Chemother..

[34] Ianevski A., Froysa I.T., Lysvand H., Calitz C., Smura T., Nilsen H.J.S., Hoyer E., Afset J.E., Sridhar A., Wolthers K.C. (2024). The combination of pleconaril, rupintrivir, and remdesivir efficiently inhibits enterovirus infections in vitro, delaying the development of drug-resistant virus variants. Antivir. Res..

[35] Thibaut H.J., Leyssen P., Puerstinger G., Muigg A., Neyts J., De Palma A.M. (2011). Towards the design of combination therapy for the treatment of enterovirus infections. Antivir. Res..

[36] Thibaut H.J., De Palma A.M., Neyts J. (2012). Combating enterovirus replication: State-of-the-art on antiviral research. Biochem. Pharmacol..

[37] Lim T.Y.M., Jaladanki C.K., Wong Y.H., Yogarajah T., Fan H., Chu J.J.H. (2024). Tanomastat exerts multi-targeted inhibitory effects on viral capsid dissociation and RNA replication in human enteroviruses. EBioMedicine.

[38] De Colibus L.G., Wang X.X., Spyrou J.A.B., Kelly J., Ren J.S., Grimes J., Puerstinger G., Stonehouse N., Walter T.S., Hu Z.Y. (2014). More-powerful virus inhibitors from structure-based analysis of HEV71 capsid-binding molecules. Nat. Struct. Mol. Biol..

[39] Lane T.R., Fu J.N., Sherry B., Tarbet B., Hurst B.L., Riabova O., Kazakova E., Egorova A., Clarke P., Leser J.S. (2023). Efficacy of an isoxazole-3-carboxamide analog of pleconaril in mouse models of Enterovirus-D68 and Coxsackie B5. Antivir. Res..

[40] Ma C.L., Hu Y.M., Zhang J.T., Wang J. (2020). Pharmacological Characterization of the Mechanism of Action of R523062, a Promising Antiviral for Enterovirus D68. ACS Infect. Dis..

[41] Tan Y.W., Yam W.K., Kooi R.J.W., Westman J., Arbrandt G., Chu J.J.H. (2021). Novel capsid binder and PI4KIIIbeta inhibitors for EV-A71 replication inhibition. Sci. Rep..

[42] Zhao D.R., Guo X.Y., Lin B.B., Huang R., Li H.Y., Wang Q., Zeng Y.L., Shang Y., Wu Y. (2024). Magnolol against enterovirus 71 by targeting Nrf2-SLC7A11-GSH pathway. Biomed. Pharmacother..

[43] Masomian M., Lalani S., Poh C.L. (2021). Molecular Docking of SP40 Peptide towards Cellular Receptors for Enterovirus 71 (EV-A71). Molecules.

[44] Tan C.W., Chan Y.F., Sim K.M., Tan E.L., Poh C.L. (2012). Inhibition of Enterovirus 71 (EV-71) Infections by a Novel Antiviral Peptide Derived from EV-71 Capsid Protein VP1. PLoS ONE.

[45] Pei Z.C., Wang H., Zhao Z.L., Chen X., Huan C., Zhang W.Y. (2022). Chemokine PF4 Inhibits EV71 and CA16 Infections at the Entry Stage. J. Virol..

[46] Lv S., Li C.Y., Pei Z.C., Hu Z.W., Du Y.N., Zheng B.S., Zhang W.Y. (2025). Platelet factor 4-derived C15 peptide broadly inhibits enteroviruses by disrupting viral attachment. J. Virol..

[47] Yang X.Y., Aloise C., van Vliet A.L.W., Zwaagstra M., Lyoo H., Cheng A.C., van Kuppeveld F.J.M. (2022). Proteolytic Activities of Enterovirus 2A Do Not Depend on Its Interaction with SETD3. Viruses.

[48] Wang Y.Y., Zou W.J., Niu Y., Wang S.Y., Chen B.T., Xiong R., Zhang P., Luo Z.J., Wu Y., Fan C.F. (2023). Phosphorylation of enteroviral 2Apro at Ser/Thr125 benefits its proteolytic activity and viral pathogenesis. J. Med. Virol..

[49] Falah N., Montserret R., Lelogeais V., Schuffenecker I., Lina B., Cortay J.C., Violot S. (2012). Blocking human enterovirus 71 replication by targeting viral 2A protease. J. Antimicrob. Chemother..

[50] Chen S.G., Cheng M.L., Chen K.H., Horng J.T., Liu C.C., Wang S.M., Sakurai H., Leu Y.L., Wang S.D., Ho H.Y. (2017). Antiviral activities of Schizonepeta tenuifolia Briq. against enterovirus 71 in vitro and in vivo. Sci. Rep..

[51] Zang L.C., Yang X.Y., Chen Y., Huang F., Yuan Y.K., Chen X.J., Zuo Y.B., Miao Y., Gu J., Guo H. (2023). Ubiquitin E3 ligase SPOP is a host negative regulator of enterovirus 71-encoded 2A protease. J. Virol..

[52] Wang C.Y., Huang A.C., Hour M.J., Huang S.H., Kung S.H., Chen C.H., Chen I.C., Chang Y.S., Lien J.C., Lin C.W. (2015). Antiviral Potential of a Novel Compound CW-33 against Enterovirus A71 via Inhibition of Viral 2A Protease. Viruses.

[53] Zhong Z.J., Zhang D.J., Peng Z.G., Li Y.H., Shan G.Z., Zuo L.M., Wu L.T., Li S.Y., Gao R.M., Li Z.R. (2013). Synthesis and antiviral activity of a novel class of (5-oxazolyl)phenyl amines. Eur. J. Med. Chem..

[54] Frost J., Rudy M.J., Leser J.S., Tan H.Z., Hu Y.M., Wang J., Clarke P., Tyler K.L. (2023). Telaprevir Treatment Reduces Paralysis in a Mouse Model of Enterovirus D68 Acute Flaccid Myelitis. J. Virol..

[55] Musharrafieh R., Ma C.L., Zhang J.T., Hu Y.M., Diesing J.M., Marty M.T., Wang J. (2019). Validating Enterovirus D68-2Apro as an Antiviral Drug Target and the Discovery of Telaprevir as a Potent D68-2A^pro^ Inhibitor. J. Virol..

[56] Tan B., Liu C., Li K., Jadhav P., Lambrinidis G., Zhu L., Olson L., Tan H.Z., Wen Y., Kolocouris A. (2023). Structure-Based Lead Optimization of Enterovirus D68 2A Protease Inhibitors. J. Med. Chem..

[57] Xie S.Q., Wang K., Yu W.J., Lu W., Xu K., Wang J.W., Ye B., Schwarz W., Jin Q., Sun B. (2011). DIDS blocks a chloride-dependent current that is mediated by the 2B protein of enterovirus 71. Cell Res..

[58] Xiang Z.C., Tian Z.Q., Wang G.Y., Liu L.L., Li K.L., Wang W.J., Lei X.B., Ren L.L., Wang J.W. (2023). CD74 Interacts with Proteins of Enterovirus D68 To Inhibit Virus Replication. Microbiol. Spectr..

[59] Yin C.H., Zhao H.M., Xia X.Y., Pan Z.Y., Li D.Q., Zhang L.L. (2024). Picornavirus 2C proteins: Structure-function relationships and interactions with host factors. Front. Cell. Infect. Microbiol..

[60] Shankar K., Sorin M.N., Sharma H., Skoglund O., Dahmane S., ter Beek J., Tesfalidet S., Nenzén L., Carlson L.A. (2024). In vitro reconstitution reveals membrane clustering and RNA recruitment by the enteroviral AAA+ ATPase 2C. PLoS Pathog..

[61] Guan H.X., Tian J., Qin B., Wojdyla J.A., Wang B., Zhao Z.D., Wang M.T., Cui S. (2017). Crystal structure of 2C helicase from enterovirus 71. Sci. Adv..

[62] Zuo J., Quinn K.K., Kye S., Cooper P., Damoiseaux R., Krogstad P. (2012). Fluoxetine Is a Potent Inhibitor of Coxsackievirus Replication. Antimicrob. Agents Chemother..

[63] Lee M.F., Tham S.K., Poh C.L. (2025). Antiviral Strategies Targeting Enteroviruses: Current Advances and Future Directions. Viruses.

[64] Messacar K., Sillau S., Hopkins S.E., Otten C., Wilson-Murphy M., Wong B., Santoro J.D., Treister A., Bains H.K., Torres A. (2019). Safety, tolerability, and efficacy of fluoxetine as an antiviral for acute flaccid myelitis. Neurology.

[65] Ulferts R., van der Linden L., Thibaut H.J., Lanke K.H.W., Leyssen P., Coutard B., De Palma A.M., Canard B., Neyts J., van Kuppeveld F.J.M. (2013). Selective Serotonin Reuptake Inhibitor Fluoxetine Inhibits Replication of Human Enteroviruses B and D by Targeting Viral Protein 2C. Antimicrob. Agents Chemother..

[66] Bauer L., Manganaro R., Zonsics B., Hurdiss D.L., Zwaagstra M., Donselaar T., Welter N.G.E., van Kleef R., Lopez M.L., Bevilacqua F. (2020). Rational design of highly potent broad-spectrum enterovirus inhibitors targeting the nonstructural protein 2C. PLoS Biol..

[67] Musharrafieh R., Zhang J.T., Tuohy P., Kitamura N., Bellampalli S.S., Hu Y.M., Khanna R., Wang J. (2019). Discovery of Quinoline Analogues as Potent Antivirals against Enterovirus D68 (EV-D68). J. Med. Chem..

[68] Sadeghipour S., Bek E.J., McMinn P.C. (2012). Selection and characterisation of guanidine-resistant mutants of human enterovirus 71. Virus Res..

[69] Klein M., Hadaschik D., Zimmermann H., Eggers H.J., Nelsen-Salz B. (2000). The picornavirus replication inhibitors HBB and guanidine in the echovirus-9 system: The significance of viral protein 2C. J. Gen. Virol..

[70] De Palma A.M., Heggermont W., Lanke K., Coutard B., Bergmann M., Monforte A.M., Canard B., De Clercq E., Chimirri A., Pürstinger G. (2008). The thiazolobenzimidazole TBZE-029 inhibits enterovirus replication by targeting a short region immediately downstream from motif C in the nonstructural protein 2C. J. Virol..

[71] Ulferts R., de Boer S.M., van der Linden L., Bauer L., Lyoo H.R., Maté M.J., Lichière J., Canard B., Lelieveld D., Omta W. (2016). Screening of a Library of FDA-Approved Drugs Identifies Several Enterovirus Replication Inhibitors That Target Viral Protein 2C. Antimicrob. Agents Chemother..

[72] Musharrafieh R., Kitamura N., Hu Y.M., Wang J. (2020). Development of broad-spectrum enterovirus antivirals based on quinoline scaffold. Bioorganic Chem..

[73] Tang Q., Xu Z.C., Jin M.Y., Shu T., Chen Y.N., Feng L.L., Zhang Q.H., Lan K., Wu S.W., Zhou H.B. (2020). Identification of dibucaine derivatives as novel potent enterovirus 2C helicase inhibitors: In vitro, in vivo, and combination therapy study. Eur. J. Med. Chem..

[74] Xing Y.P., Zuo J., Krogstad P., Jung M.E. (2018). Synthesis and Structure-Activity Relationship (SAR) Studies of Novel Pyrazolopyridine Derivatives as Inhibitors of Enterovirus Replication. J. Med. Chem..

[75] Li K., Rudy M.J., Hu Y., Tan H., Lambrinidis G., Wu X., Georgiou K., Tan B., Frost J., Wilson C. (2025). A rationally designed 2C inhibitor prevents enterovirus D68-infected mice from developing paralysis. Nat. Commun..

[76] Li D.Q., Zhang L.L. (2022). Structure Prediction and Potential Inhibitors Docking of Enterovirus 2C Proteins. Front. Microbiol..

[77] Fang Y., Wang C., Wang C., Yang R.Y., Bai P., Zhang X.Y., Kong J., Yin L., Qiu Y., Zhou X. (2021). Antiviral Peptides Targeting the Helicase Activity of Enterovirus Nonstructural Protein 2C. J. Virol..

[78] Li X.H., Wang M.S., Cheng A.C., Wen X.J., Ou X.M., Mao S., Gao Q., Sun D., Jia R.Y., Yang Q. (2020). Enterovirus Replication Organelles and Inhibitors of Their Formation. Front. Microbiol..

[79] Yang H., Fan T.T., Xun M., Wu B., Guo S.R., Li X.Y., Zhao X.H., Yao H.Y., Wang H.L., Heise M.T. (2024). N-terminal acetyltransferase 6 facilitates enterovirus 71 replication by regulating PI4KB expression and replication organelle biogenesis. J. Virol..

[80] Xiao X., Lei X.B., Zhang Z.Z., Ma Y.J., Qi J.L., Wu C., Xiao Y., Li L., He B., Wang J.W. (2017). Enterovirus 3A Facilitates Viral Replication by Promoting Phosphatidylinositol 4-Kinase IIIβ-ACBD3 Interaction. J. Virol..

[81] Delang L., Harak C., Benkheil M., Khan H., Leyssen P., Andrews M., Lohmann V., Neyts J. (2018). PI4KIII inhibitor enviroxime impedes the replication of the hepatitis C virus by inhibiting PI3 kinases. J. Antimicrob. Chemother..

[82] Arita M., Kojima H., Nagano T., Okabe T., Wakita T., Shimizu H. (2011). Phosphatidylinositol 4-Kinase III Beta Is a Target of Enviroxime-Like Compounds for Antipoliovirus Activity. J. Virol..

[83] Albulescu L., Bigay J., Biswas B., Weber-Boyvat M., Dorobantu C.M., Delang L., van der Schaar H.M., Jung Y.S., Neyts J., Olkkonen V.M. (2017). Uncovering oxysterol-binding protein (OSBP) as a target of the anti-enteroviral compound TTP-8307. Antivir. Res..

[84] Sála M., Kögler M., Placková P., Mejdrová I., Hrebabecky H., Procházková E., Strunin D., Lee G., Birkus G., Weber J. (2016). Purine analogs as phosphatidylinositol 4-kinase IIIβ inhibitors. Bioorganic Med. Chem. Lett..

[85] Arita M., Dobrikov G., Pürstinger G., Galabov A.S. (2017). Allosteric Regulation of Phosphatidylinositol 4-Kinase Ill Beta by an Antipicornavirus Compound MDL-860. ACS Infect. Dis..

[86] Vassileva-Pencheva R., Galabov A.S. (2016). Effectiveness of the Consecutive Alternating Administration Course of a Triple Antiviral Combination in Coxsackievirus B3 Infections in Mice. Drug Res..

[87] Laajala M., Zwaagstra M., Martikainen M., Nekoua M.P., Benkahla M., Sane F., Gervais E., Campagnola G., Honkimaa A., Sioofy-Khojine A.B. (2023). Vemurafenib Inhibits Acute and Chronic Enterovirus Infection by Affecting Cellular Kinase Phosphatidylinositol 4-Kinase Type IIIβ. Microbiol. Spectr..

[88] Butrym M., Byvald F., Blanter M., Ringqvist E.E., Vasylovska S., Marjomäki V., Lau J., Stone V.M., Flodström-Tullberg M. (2024). Vemurafenib inhibits the replication of diabetogenic enteroviruses in intestinal epithelial and pancreatic beta cells. Antivir. Res..

[89] Vergez I., Nekoua M.P., Arbrandt G., Westman J., Alidjinou E.K., Hober D. (2024). Macrophages can transmit coxsackievirus B4 to pancreatic cells and can impair these cells. J. Med. Virol..

[90] Cheong D.H.J., Yogarajah T., Wong Y.H., Arbrandt G., Westman J., Chu J.J.H. (2023). CUR-N399, a PI4KB inhibitor, for the treatment of Enterovirus A71 infection. Antivir. Res..

[91] Albulescu L., Strating J., Thibaut H.J., van der Linden L., Shair M.D., Neyts J., van Kuppeveld F.J.M. (2015). Broad-range inhibition of enterovirus replication by OSW-1, a natural compound targeting OSBP. Antivir. Res..

[92] Shen K.W., Jin X.L. (2025). OSW-1 inhibits tumor cell proliferation and migration via uncoupling protein 2 in hepatocellular carcinoma. Oncol. Lett..

[93] Gao Q.Q., Yuan S.L., Zhang C., Wang Y., Wang Y.Z., He G.M., Zhang S.Y., Altmeyer R., Zou G. (2015). Discovery of Itraconazole with Broad-Spectrum In Vitro Antienterovirus Activity That Targets Nonstructural Protein 3A. Antimicrob. Agents Chemother..

[94] Roberts B.L., Severance Z.C., Bensen R.C., Le-McClain A.T., Malinky C.A., Mettenbrink E.M., Nuñez J.I., Reddig W.J., Blewett E.L., Burgett A.W.G. (2019). Differing activities of oxysterol-binding protein (OSBP) targeting anti-viral compounds. Antivir. Res..

[95] Ren X.J., Yin M.G., Zhao Q.Q., Zheng Z.X., Wang H.Y., Lu Z.J., Li X.M., Qian P. (2023). Foot-and-Mouth Disease Virus Induces Porcine Gasdermin E-Mediated Pyroptosis through the Protease Activity of 3Cpro. J. Virol..

[96] Brimble M.A., Stubbing L.A., Hermant Y.O., Yang S.H., Hubert J.G., Pearl E.S., McSweeney A.M., Young V.L., Campbell A.C., Opel-Reading H.K. (2025). Broad-Spectrum Peptidomimetic Inhibitors of Norovirus and Coronavirus 3C-like Proteases. ACS Infect. Dis..

[97] Yu W.D., Jin Q.Y., Zeng M.S., Liu J.Y., Xu P.P. (2022). Geraniin as a potential inhibitor of SARS-CoV-2 3CLpro. Nat. Prod. Res..

[98] Wang J., Hu Y.M., Zheng M. (2022). Enterovirus A71 antivirals: Past, present, and future. Acta Pharm. Sin. B.

[99] Le T.T.V., Do P.C. (2022). Molecular docking study of various Enterovirus-A71 3C protease proteins and their potential inhibitors. Front. Microbiol..

[100] Wang S.Q., Pang Z.H., Fan H.H., Tong Y.G. (2024). Advances in anti-EV-A71 drug development research. J. Adv. Res..

[101] Lu G.W., Qi J.X., Chen Z.J., Xu X., Gao F., Lin D.Z., Qian W.K., Liu H., Jiang H.L., Yan J.H. (2011). Enterovirus 71 and Coxsackievirus A16 3C Proteases: Binding to Rupintrivir and Their Substrates and Anti-Hand, Foot, and Mouth Disease Virus Drug Design. J. Virol..

[102] Lee J., Lee H.L., Kim H., Gil Y., Lee S.H., Jung Y.S., Shin J.S., Jo I. (2026). Structural insights into the antiviral efficacy of AG7404 against human rhinovirus 3C proteases. IUCrJ.

[103] Rhoden E., Liu H.M., Wang-Chern S.W., Oberste M.S. (2013). Anti-poliovirus activity of protease inhibitor AG-7404, and assessment of in vitro activity in combination with antiviral capsid inhibitor compounds. Antivir. Res..

[104] Ravlo E., Ianevski A., Schjolberg J.O., Solvang V., Dumaru R., Lysvand H., Hankinson J., Vähä-Koskela M., Vainionpää S., Varhe A. (2025). Synergistic combination of orally available safe-in-man pleconaril, AG7404, and mindeudesivir inhibits enterovirus infections in human cell and organoid cultures. Cell. Mol. Life Sci..

[105] Zhai Y., Ma Y., Ma F., Nie Q., Ren X., Wang Y., Shang L., Yin Z. (2016). Structure–activity relationship study of peptidomimetic aldehydes as enterovirus 71 3C protease inhibitors. Eur. J. Med. Chem..

[106] Wang Y.X., Cao L., Zhai Y.Y., Yin Z., Sun Y.N., Shang L.Q. (2017). Structure of the Enterovirus 71 3C Protease in Complex with NK-1.8k and Indications for the Development of Antienterovirus Protease Inhibitor. Antimicrob. Agents Chemother..

[107] Tijsma A., Franco D., Tucker S., Hilgenfeld R., Froeyen M., Leyssen P., Neyts J. (2014). The Capsid Binder Vapendavir and the Novel Protease Inhibitor SG85 Inhibit Enterovirus 71 Replication. Antimicrob. Agents Chemother..

[108] Tan J.Z., George S., Kusov Y., Perbandt M., Anemüller S., Mesters J.R., Norder H., Coutard B., Lacroix C., Leyssen P. (2013). 3C Protease of Enterovirus 68: Structure-Based Design of Michael Acceptor Inhibitors and Their Broad-Spectrum Antiviral Effects against Picornaviruses. J. Virol..

[109] Zhai Y.Y., Zhao X.S., Cui Z.J., Wang M., Wang Y.X., Li L.F., Sun Q., Yang X., Zeng D.B., Liu Y. (2015). Cyanohydrin as an Anchoring Group for Potent and Selective Inhibitors of Enterovirus 71 3C Protease. J. Med. Chem..

[110] Ma Y.Y., Shang C.Y., Yang P., Li L.F., Zhai Y.Y., Yin Z., Wang B.H., Shang L.Q. (2018). 4-Iminooxazolidin-2-one as a Bioisostere of the Cyanohydrin Moiety: Inhibitors of Enterovirus 71 3C Protease. J. Med. Chem..

[111] Xu B.H., Liu M.J., Ma S., Ma Y.Y., Liu S., Shang L.Q., Zhu C., Ye S., Wang Y.X. (2021). 4-Iminooxazolidin-2-One as a Bioisostere of Cyanohydrin Suppresses EV71 Proliferation by Targeting 3Cpro. Microbiol. Spectr..

[112] Ma G.H., Ye Y., Zhang D., Xu X., Si P., Peng J.L., Xiao Y.L., Cao R.Y., Yin Y.L., Chen J. (2016). Identification and biochemical characterization of DC07090 as a novel potent small molecule inhibitor against human enterovirus 71 3C protease by structure-based virtual screening. Eur. J. Med. Chem..

[113] Kim Y., Lovell S., Tiew K.C., Mandadapu S.R., Alliston K.R., Battaile K.P., Groutas W.C., Chang K.O. (2012). Broad-Spectrum Antivirals against 3C or 3C-Like Proteases of Picornaviruses, Noroviruses, and Coronaviruses. J. Virol..

[114] Li P., Wu S.Q., Xiao T.Y.C., Li Y.L., Su Z.M., Wei W., Hao F., Hu G.P., Lin F.S., Chen X.S. (2020). Design, synthesis, and evaluation of a novel macrocyclic anti-EV71 agent. Bioorganic Med. Chem..

[115] Yao C.G., Xi C.L., Hu K.H., Gao W., Cai X.F., Qin J.L., Lv S.Y., Du C.H., Wei Y.H. (2018). Inhibition of enterovirus 71 replication and viral 3C protease by quercetin. Virol. J..

[116] Liu Y., Wang C., Tong H., Zhou X., Fang Y. (2025). Peptides designed based on 3C substrates exhibit antiviral efficacy in vivo. Antivir. Res..

[117] Wei Y.H., Liu H.H., Hu D., He Q., Yao C.G., Li H.L., Hu K.H., Wang J. (2024). Recent Advances in Enterovirus A71 Infection and Antiviral Agents. Lab. Investig..

[118] Xu N., Yang J., Zheng B.S., Zhang Y., Cao Y.M., Huan C., Wang S.Q., Chang J.B., Zhang W.Y. (2020). The Pyrimidine Analog FNC Potently Inhibits the Replication of Multiple Enteroviruses. J. Virol..

[119] Radoshitzky S.R., Iversen P., Lu X.H., Zou J., Kaptein S.J.F., Stuthman K.S., Van Tongeren S.A., Steffens J., Gong R.Y., Truong H. (2023). Expanded profiling of Remdesivir as a broad-spectrum antiviral and low potential for interaction with other medications in vitro. Sci. Rep..

[120] Bartoletti M., Mozaffari E., Amin A.N., Doi Y., Loubet P., Rivera C.G., Roshon M., Rawal A., Kaiser E., Nicolae M.V. (2025). A Systematic Review and Meta-analysis of the Effectiveness of Remdesivir to Treat Severe Acute Respiratory Syndrome Coronavirus 2 in Hospitalized Patients: Have the Guidelines Evolved With the Evidence?. Clin. Infect. Dis..

[121] Ye W., Yao M., Dong Y.C., Ye C.T., Wang D., Liu H., Ma H.W., Zhang H., Qi L.B., Yang Y.W. (2020). Remdesivir (GS-5734) Impedes Enterovirus Replication Through Viral RNA Synthesis Inhibition. Front. Microbiol..

[122] Stögbauer J., Schegerer V., Berger F.K., Schulz-Schaeffer W., Fassbender K., Naumann J., Smola S., Eisenbeis J., Bewarder M., Rosar F. (2025). Severe coxsackie virus B5 encephalitis mimics autoimmune limbic encephalitis in a young woman under long-term B-cell depletion with ocrelizumab: A case report. Mult. Scler. J..

[123] Abdelnabi R., de Morais A.T.S., Leyssen P., Imbert I., Beaucourt S., Blanc H., Froeyen M., Vignuzzi M., Canard B., Neyts J. (2017). Understanding the Mechanism of the Broad-Spectrum Antiviral Activity of Favipiravir (T-705): Key Role of the F1 Motif of the Viral Polymerase. J. Virol..

[124] Deng C.L., Yeo H.M., Ye H.Q., Liu S.Q., Shang B.D., Gong P., Alonso S., Shi P.Y., Zhang B. (2014). Inhibition of Enterovirus 71 by Adenosine Analog NITD008. J. Virol..

[125] Chen T.C., Chang H.Y., Lin P.F., Chern J.H., Hsu J.T.A., Chang C.Y., Shih S.R. (2009). Novel Antiviral Agent DTriP-22 Targets RNA-Dependent RNA Polymerase of Enterovirus 71. Antimicrob. Agents Chemother..

[126] van der Linden L., Vives-Adrián L., Selisko B., Ferrer-Orta C., Liu X.R., Lanke K., Ulferts R., De Palma A.M., Tanchis F., Goris N. (2015). The RNA Template Channel of the RNA-Dependent RNA Polymerase as a Target for Development of Antiviral Therapy of Multiple Genera within a Virus Family. PLoS Pathog..

[127] Velu A.B., Chen G.W., Hsieh P.T., Horng J.T., Hsu J.T.A., Hsieh H.P., Chen T.C., Weng K.F., Shih S.R. (2014). BPR-3P0128 inhibits RNA-dependent RNA polymerase elongation and VPg uridylylation activities of Enterovirus 71. Antivir. Res..

[128] Hung H.C., Chen T.C., Fang M.Y., Yen K.J., Shih S.R., Hsu J.T.A., Tseng C.P. (2010). Inhibition of enterovirus 71 replication and the viral 3D polymerase by aurintricarboxylic acid. J. Antimicrob. Chemother..

[129] Dai W.W., Wu Y., Bi J.P., Lu X.T., Hou A., Zhou Y., Sun B., Kong W., Barbier J., Cintrat J.C. (2017). Antiviral effects of Retro-2cycl and Retro-2.1 against Enterovirus 71 in vitro and in vivo. Antivir. Res..

[130] Abdullah S.W., Wu J.E., Wang X.F., Guo H.C., Sun S.Q. (2023). Advances and Breakthroughs in IRES-Directed Translation and Replication of Picornaviruses. mBio.

[131] Martinez-Salas E., Francisco-Velilla R., Fernandez-Chamorro J., Embarek A.M. (2018). Insights into Structural and Mechanistic Features of Viral IRES Elements. Front. Microbiol..

[132] Tang Q., Li S.L., Du L.Q., Chen S.H., Gao J.Y., Cai Y., Xu Z.C., Zhao Z.X., Lan K., Wu S.W. (2020). Emetine protects mice from enterovirus infection by inhibiting viral translation. Antivir. Res..

[133] Liu H.K., Fan W.Z., Li H.Q., Qiao L.L., Liu Z.L., Zhu B.W., Guo J., Huang K., Tang Y.Y., Wen J. (2025). Idarubicin versus Epirubicin in Transarterial Chemoembolization for Barcelona Clinic Liver Cancer Stage B Hepatocellular Carcinoma: An Open-label, Randomized, Phase IV Trial. Radiology.

[134] Hou H.Y., Lu W.W., Wu K.Y., Lin C.W., Kung S.H. (2016). Idarubicin is a broad-spectrum enterovirus replication inhibitor that selectively targets the virus internal ribosomal entry site. J. Gen. Virol..

[135] Pourianfar H.R., Poh C.L., Fecondo J., Grollo L. (2012). In vitro evaluation of the antiviral activity of heparan sulfate mimetic compounds against Enterovirus 71. Virus Res..

[136] Gunaseelan S., Wong K.Z., Min N., Sun J., Ismail N., Tan Y.J., Lee R.C.H., Chu J.J.H. (2019). Prunin suppresses viral IRES activity and is a potential candidate for treating enterovirus A71 infection. Sci. Transl. Med..

[137] Chuang Y.T., Lin Y.L., Lin J.Y. (2024). Licochalcone A regulates viral IRES activity to inhibit enterovirus replication. Antivir. Res..

[138] Dai W.W., Bi J.P., Li F., Wang S., Huang X.Y., Meng X.Y., Sun B., Wang D.L., Kong W., Jiang C.L. (2019). Antiviral Efficacy of Flavonoids against Enterovirus 71 Infection in Vitro and in Newborn Mice. Viruses.

[139] Davila-Calderon J., Patwardhan N.N., Chiu L.Y., Sugarman A., Cai Z.G., Penutmutchu S.R., Li M.L., Brewer G., Hargrove A.E., Tolbert B.S. (2020). IRES-targeting small molecule inhibits enterovirus 71 replication via allosteric stabilization of a ternary complex. Nat. Commun..

[140] Seclén E., González M.D., Lapaz M., Rodríguez C., del Romero J., Aguilera A., de Mendoza C., Soriano V., Poveda E. (2010). Primary resistance to maraviroc in a large set of R5-V3 viral sequences from HIV-1-infected patients. J. Antimicrob. Chemother..

[141] Müller C., Schulte F.W., Lange-Grünweller K., Obermann W., Madhugiri R., Pleschka S., Ziebuhr J., Hartmann R.K., Grünweller A. (2018). Broad-spectrum antiviral activity of the eIF4A inhibitor silvestrol against corona- and picornaviruses. Antivir. Res..

[142] Peron G., Mastinu A., Peña-Corona S.I., Hernández-Parra H., Leyva-Gómez G., Calina D., Sharifi-Rad J. (2024). Silvestrol, a potent anticancer agent with unfavourable pharmacokinetics: Current knowledge on its pharmacological properties and future directions for the development of novel drugs. Biomed. Pharmacother..

[143] Too I.H.K., Bonne I., Tan E.L., Chu J.J.H., Alonso S. (2018). Prohibitin plays a critical role in Enterovirus 71 neuropathogenesis. PLoS Pathog..

[144] Yuan S.F., Chu H., Huang J.J., Zhao X.Y., Ye Z.W., Lai P.M., Wen L., Cai J.P., Mo Y.F., Cao J.L. (2020). Viruses harness Yxxempty set motif to interact with host AP2M1 for replication: A vulnerable broad-spectrum antiviral target. Sci. Adv..

[145] Liang Y.C., Kong Y., Rao M.L., Zhou X., Li C.C., Meng Y., Chen Y.X., Li H.J., Luo Z. (2024). Inhibition of ESCRT-independent extracellular vesicles biogenesis suppresses enterovirus 71 replication and pathogenesis in mice. Int. J. Biol. Macromol..

[146] Bieganowski P., Dalidowska I., Gazi O., Guzowska M., Przybylski M. (2025). Study of Hsp90α and Hsp90β role in virus replication using cell lines with Hsp90 gene knockouts. Virus Genes.

[147] van der Linden L., Wolthers K.C., van Kuppeveld F.J.M. (2015). Replication and Inhibitors of Enteroviruses and Parechoviruses. Viruses.

[148] Yang Y.Q., Cao L., Gao H.Y., Wu Y., Wang Y.X., Fang F., Lan T.L., Lou Z.Y., Rao Y. (2019). Discovery, Optimization, and Target Identification of Novel Potent Broad-Spectrum Antiviral Inhibitors. J. Med. Chem..

[149] Yang Q.Y., Wu C.Y., Zhu G.Y., Ren F.L., Lin B.B., Huang R., Hu X.J., Zhao D.R., Peng K., Wang Q.Y. (2023). ML390 inhibits enterovirus 71 replication by targeting de novo pyrimidine biosynthesis pathway. Antivir. Res..

[150] Fu H., Zhang Z., Dai Y., Liu S.M., Fu E.Q. (2020). Brequinar inhibits enterovirus replication by targeting biosynthesis pathway of pyrimidines. Am. J. Transl. Res..

[151] Zhang L., Das P., Schmolke M., Manicassamy B., Wang Y.M., Deng X.Y., Cai L., Tu B.P., Forst C.V., Roth M.G. (2012). Inhibition of pyrimidine synthesis reverses viral virulence factor-mediated block of mRNA nuclear export. J. Cell Biol..

[152] Zhang X., Cao Y., Liu Y., Deng S., Lv S., Peng W., Fang L., Zhang L., Wu Y., Yu L. (2026). Regorafenib, a multitargeted kinase inhibitor, inhibits enterovirus 71 infection by suppressing the MAPK pathway. Int. J. Antimicrob. Agents.

[153] Wang L., Xie W., Zhang L., Li D.F., Yu H., Xiong J.Z., Peng J., Qiu J., Sheng H.L., He X.M. (2018). CVB3 Nonstructural 2A Protein Modulates SREBP1a Signaling via the MEK/ERK Pathway. J. Virol..

[154] Johansen L.M., Brannan J.M., Delos S.E., Shoemaker C.J., Stossel A., Lear C., Hoffstrom B.G., DeWald L.E., Schornberg K.L., Scully C. (2013). FDA-Approved Selective Estrogen Receptor Modulators Inhibit Ebola Virus Infection. Sci. Transl. Med..

[155] Jurgeit A., McDowell R., Moese S., Meldrum E., Schwendener R., Greber U.F. (2012). Niclosamide Is a Proton Carrier and Targets Acidic Endosomes with Broad Antiviral Effects. PLoS Pathog..

[156] Chen J.Y., Zhao T.Q., Ji M., Xia B.H. (2025). Antiviral candidates for enterovirus 71: Targeting viral proteome and stage-specific lifecycle interventions. Virol. J..

[157] Torchilin V.P. (2014). Multifunctional, stimuli-sensitive nanoparticulate systems for drug delivery. Nat. Rev. Drug Discov..

